# Hydrometeorological characterization and estimation of landfill leachate generation in the Eastern Amazon/Brazil

**DOI:** 10.7717/peerj.14686

**Published:** 2023-01-23

**Authors:** Carlos Armando Reyes Flores, Helenilza Ferreira Albuquerque Cunha, Alan Cavalcanti da Cunha

**Affiliations:** 1 Postgraduate Program in Tropical Biodiversity, Federal University of Amapá, Macapá, Amapá, Brazil; 2 Postgraduate Program in Environmental Sciences, Federal University of Amapá, Macapá, Amapá, Brazil; 3 Environment and Development Department, Federal University of Amapá, Macapá, Amapá, Brazil; 4 Civil Engineering Department, Federal University of Amapá, Macapá, Amapá, Brazil

**Keywords:** Hydric balance, Precipitation, Flow, Leachate, Evaporation, Hydrological processes

## Abstract

The complex physical-chemical and microbiological composition of leachate in sanitary landfills sets the adequate treatment for different waste types. However, before the final disposal of wastes in receptor waterbodies, it is essential to use specific methods to quantitatively prevent internal flows to find proper treatments. The aim of the present research is to use hydrological models to estimate monthly leachate flow generation in Macapá’s Municipal Sanitary Landfill (ASMM), Amapá State, Brazil. Disregarding the temporal trend bias, the average (0.45 m^3^s^−1^), minimum (0.07 m^3^s^−1^) and maximum (0.72 m^3^s^−1^) flows were estimated based on hydrological models in the literature (R_max_ = 99%, *p* < 0.05). The results estimated from different hydrological gauges pointed towards significant spatial variations in final discharge. Thus, estimated flows worked as reference to calculate the loads of coproducts and nutrients concerning different operational stages in ASMM. Therefore, rain intensity estimates have pointed out precipitation variability, and it has significantly affected leachate flow. In conclusion, there would be a proportional increase in leachate flow during extreme maximum precipitation events; overflow would be the effect of such flows and it would assumingly have impact on its surrounding areas. It is also possible estimating some degree of rainfall impact over ASMM’s infrastructure in the long term (>10 years), since it could influence its lifespan.

## Introduction

The lack of a selective collection system in Macapá County, Amapá State, Brazil, accounts for the fact that all sorts of solid residues are inappropriate and are simultaneously disposed in the same cells of Macapá’s Municipal Sanitary Landfill (ASMM). Furthermore, nowadays, ASMM is simultaneously serving three of the sixteen municipalities in the state, namely: Macapá (522,357 estimated people), Santana (104,808 estimated people) and Mazagão (22,468 estimated people) (Instituto Brasileiro de Geografia e Estatística (IBGE), 2021). The service has been provided under a precarious cooperation agreement (management consortium) among the municipalities (Koutsoyiannis, 2003; Prabhakar et al., 2019); efforts were made in order to avoid Santana and Mazagão to be sued for not following the technical and maintenance standards in their outdoor dumps. This initiative seems to have had significant impact on the use-volume rate recorded for ASMM’s cells, a fact that likely reduced its lifespan (Huang et al., 2001; França & Ruaro, 2009).

ASMM (RumusEngenharia, 2018; Santos et al., 2019) is the main infrastructure for the final proper environmental destination of urban solid waste (USW) in Amapá State/Brazil. However, liquid waste (leachate) generation in ASMM, as well as in other similar stations in the Amazon, was never assessed in order to help better understanding its role due to operational challenges, mainly to support management actions taken to control its effects on storage cells, as well as to help better understanding its potential environmental externalities (neighborhoods) (Watzinger et al., 2006; Naveen & Malik, 2019; Sharma, Ganguly & Kumar Gupta, 2020; Li et al., 2021). Therefore, its construction is part of a national strategy substantiated by the legislation; it aims the ultimate eradication of outdoor dumps in thousands of municipalities countrywide (Ministry of the Environment of Brazil, 2020). Nevertheless, little is known about the potential operational and environmental impacts of this new USW generation rate on leachate formation in ASMM. It is so, because of the segregation of different waste types in aligned cells and of solid waste decomposition in ASMM, which prevail in the anaerobic phase and generate slurry, besides biogas. In other words, in case of precipitation over storage cells, part of the waste infiltrates in the landfill mass and part of it runs off it, regardless of all care actions taken during the landfill’s execution operational phase (Barros, 2013).

Microbiological decomposition of the waste mass landfilled in ASMM produces gases, as well as the referred dark liquid known as leachate, which has high polluting power. Leachate can be considered a more complex issue in comparison to gases produced in the organic matter decomposition process (Além Sobrinho, Povinelli & Gomes, 2009). However, the most evident danger to ASMM is the potential risk of polluting and contaminating ground and surface water within the landfill and surrounding areas. For instance, the greater the fraction of leachate that enters the ASMM, percolating and mixing with the leachate produced in the various layers of compacted solid waste in the landfill, the greater this risk. Leachate can be defined as a harmful mineralized liquid capable of transporting bacterial pollutants (Botkin & Keller, 2011). Besides the produced amount, water flow in USW sanitary landfills, which is known for not being uniform, is influenced by the main routes within the deposited USW mass (Obladen, Obladen & Barros, 2009), whose use lies on extracting gases and liquids deriving from waste decomposition (Portella & Ribeiro, 2014; Cardoso, 2021).

ASMM has been subjected to leachate recirculation system applications (Rumos Engenharia Ambiental LTDA, 2018). Thus, leachate hydraulic behavior in sanitary landfills can be taken under two heterogeneous regime typologies, which are useful for the application of water flow predictive models through the deposited USW mass: (a) domain over the high hydraulic conductivity channel and (b) slow water move matrix, with high water retention capacity (Barros, 2013).

Therefore, by using the concept addressed above, leachate flow estimates have been applied in countries such as Denmark, where Poulsen, Møoldrup (Prabhakar et al., 2019) have applied a model based on global hydric balance for 13 years of leachate generation in a sanitary landfill (Tozetto, 2008). Oftentimes, leachate volume in Brazil and in Latin America, has been determined based on the hydric balance method (Padilla, 2007). However, these studies remain scarce in the Amazon, mainly because of precarious long-term hydrometeorological data series (Flores, Cunha & Cunha, 2022).

Several mathematical models have been developed in order to predict leachate production based on basic hydrological parameters (Padilla, 2007; Tozetto, 2008; Feldhaus, 2019). These quantitative leachate predictions have been performed through mass balance, based on the following methods (Barros, 2013): Swiss, rational and general. Based on the Swiss method estimates, a fraction of the precipitation infiltrates the waste and reaches the base waterproofing layer; consequently, it must be drained. With respect to highly compacted landfills (specific weigh *ρ*_e_ > 0.7 t m^−3^), estimates show leachate production ranging from 15% to 25% (*K* = 0.15−0.25) out of the mean annual precipitation recorded for the landfill area. Based on the rational method, the surface runoff calculation for leaching is carried out based on three parameters: contribution basin area; rainfall intensity and duration; and runoff coefficient. The general method (hydric balance) allows quantifying the leaching liquids in a landfill by considering water input and output in the waste mass, during a given period-of-time (Feldhaus, 2019)

Precipitation is the main component of the hydrological cycle, it works as input in the hydrographic basins’ system, whereas its disposal—with a given amount of surface water and groundwater—is the output (Fattorelli & Fernandez, 2011). Leachate disposal in sanitary landfills is featured by random and stochastic variables, and drainage is a deterministic phenomenon (Finkler, 2012; Jayanti, 2020).

Because mean precipitation is also variable, and capable of changing extreme events that have impact on their historical series (Demirdjian, Zhou & Huffman, 2018; Li et al., 2020), it can exceed the transportation ability of drainage networks within the basins (Pagliara et al., 1998), mainly when it comes to mean precipitations. However, the opposite is observed when one finds extreme annual precipitation reduction (Balistrocchi & Grossi, 2020), as it accounts for significantly reducing the discharge peaks and for increasing the evaporation rates.

The amount of produced slurry, as a first approximation of leachate estimates in USW sanitary landfills, can be considered proportional to the percolation water volume through the USW landfill (Barros, 2013). Furthermore, the volume of sludge produced can present imminent risks to the environment for the following reasons: (a) the sludge can severely impact water quality during the transport process in critical sections of the sub-basin, especially at the final disposal site; (b) currently, the environmental license for the operation of the sanitary landfill is expired and without due inspection; the sanitary landfill is operating outside the legal normality.

It is possible using local precipitation modeling, focused on different return periods (RT = 25 years, it is the most often used in the national literature) to estimate the amount of leachate in a sanitary landfill; it is essential predicting likely extreme events to make decisions to prevent impacts (overflows) on ASMM. These impacts can help increasing the frequency and severity of floods, landslides and the disposal of untreated polluting loads in waterbodies in the surrounding areas or in groundwater (Revi et al., 2014; Salerno, Viviano & Tartari, 2018; Balistrocchi & Grossi, 2020), because they often exceed the landfill’s natural or projected volume (Pagliara et al., 1998; Barros, 2013). Therefore, it is essential to understand how extreme hydrological events can be associated with operational and leachate management problems in landfills, mainly in developing countries or in the ones with low monitoring cover or capacity (Lucena et al., 2016; Zhou et al., 2017; Lyu et al., 2018; Li et al., 2020), typical of the Brazilian Northern Region.

Different interpolation methods found in spatial precipitation models consider rain events spatially distributed within a given region, where historical data series can derive from the record of different rainfall gauges to approach the leachate production topic and estimates (Shi et al., 2019).

This way, it is necessary analyzing precipitation and climatic variables, such as minimum (T_min_) and maximum (T_max_) temperature, relative humidity (Ur), wind speed (Wv), solar radiation (Rs) and potential evapotranspiration (ETo) (Koutsoyiannis, 2003; El-Sadek et al., 2011; López, Delgado & Campo, 2018). These factors, in association with the morphometric analysis of the assessed site, allow generating the mean flow that, in its turn, enables finding the maximum flow in rainy periods (Boyle et al., 2001; Paik et al., 2005; Jayanti, 2020). This procedure makes it possible making a detailed presentation of capture processes’ spatial variability (Dal Molin et al., 2020).

There is interaction between precipitation variables and terrain features (sanitary landfill) in the sub-basin, which converge to each other in order to produce leachate (Aladejana & Fagbohun, 2019). Therefore, the solute transport velocity in generic studies increases due to the discharge flow (Covino, McGlynn & Mallard, 2011; Bergstrom et al., 2016).

An environmental slurry production scenario was elaborated based on the description of liquid waste or slurry generation in the ASMM (including its estimates) as well as theoretical hydrological estimates of water flows (average monthly precipitation or rainfall in the sub-basin). This scenario can be replicated in any similar system in rern Amazon with similar climate, where it is also not frequent finding long temporal series (>10 years). For example, the influence of precipitation on the deterioration of the infrastructure and useful life of the landfill was not evaluated. In summary, similar research on the topic has not yet been reported or carried out in the region to date. This fact is also probably related to the eminent lack of investments in the solid waste management sector in recent decades, not only in the State of Amapá, but also throughout the International Amazon (Latin America) (Flores, Cunha & Cunha, 2022).

The aim of the present research was to analyze the potential effects of precipitation on leachate generation, mainly those related to mean and maximum precipitation events, which are proportional to, and depend on, return time (RT)—they vary up by 100% when they are relatively compared to the 2 and 100 years return time (RT = 2 years, RT = 100 years), respectively. The specific goals were (a) to generate theoretical water runoff for one average typical climatic year; (b) to estimate the theoretical water runoff of the rainy season, including the drainage sub-basin ASMM is inserted in; and (c) to estimate leachate runoff, in order to represent a fraction of the water runoff, based on empirical models available in the literature on tropical climates (Padilla, 2007; Souza, 2011). In this case, the correlation coefficients were taken as good potential indicators, or as indicators of significant differences in changes in the temporal trends of precipitation historical series that influence hydrological processes, leachate generation and fraction runoff in ASMM.

An exclusive aspect of the present study lies on considering seasonal maximum peaks in slurry production in this location as the most relevant hydrological parameter to estimate leachate (Barros, 2012), in a locale or site with extreme shortage of information on this, and other, similar projects in the region.

The present study represents the first specific basic reference on mass balances (physical chemical) applied to the evaluation of leachate flows from sanitary landfills in the Amazon. Because, even in large urban centers in the Amazon, the extreme scarcity of information about these flows is notorious, particularly in the long-term analysis. Thus, although the present hydrological analysis is not exactly a scientific novelty, its focal and regional analysis is a novelty. Therefore, it can be useful as a basic technical subsidy for water monitoring around the sanitary landfill. The proposed mass balance also favors the analysis of processes that depend on this monitoring and flow estimates in places where there is a notorious scarcity of information, being necessary for both pre-, during and post-operational conditions (closing) (Ferronato & Torretta, 2019; Vaverková, 2019). Moreover, to the best of our knowledge, the present research is the first of its kind applied to the Amazon.

## Materials & Methods

### Study sites

**Figure 1:**
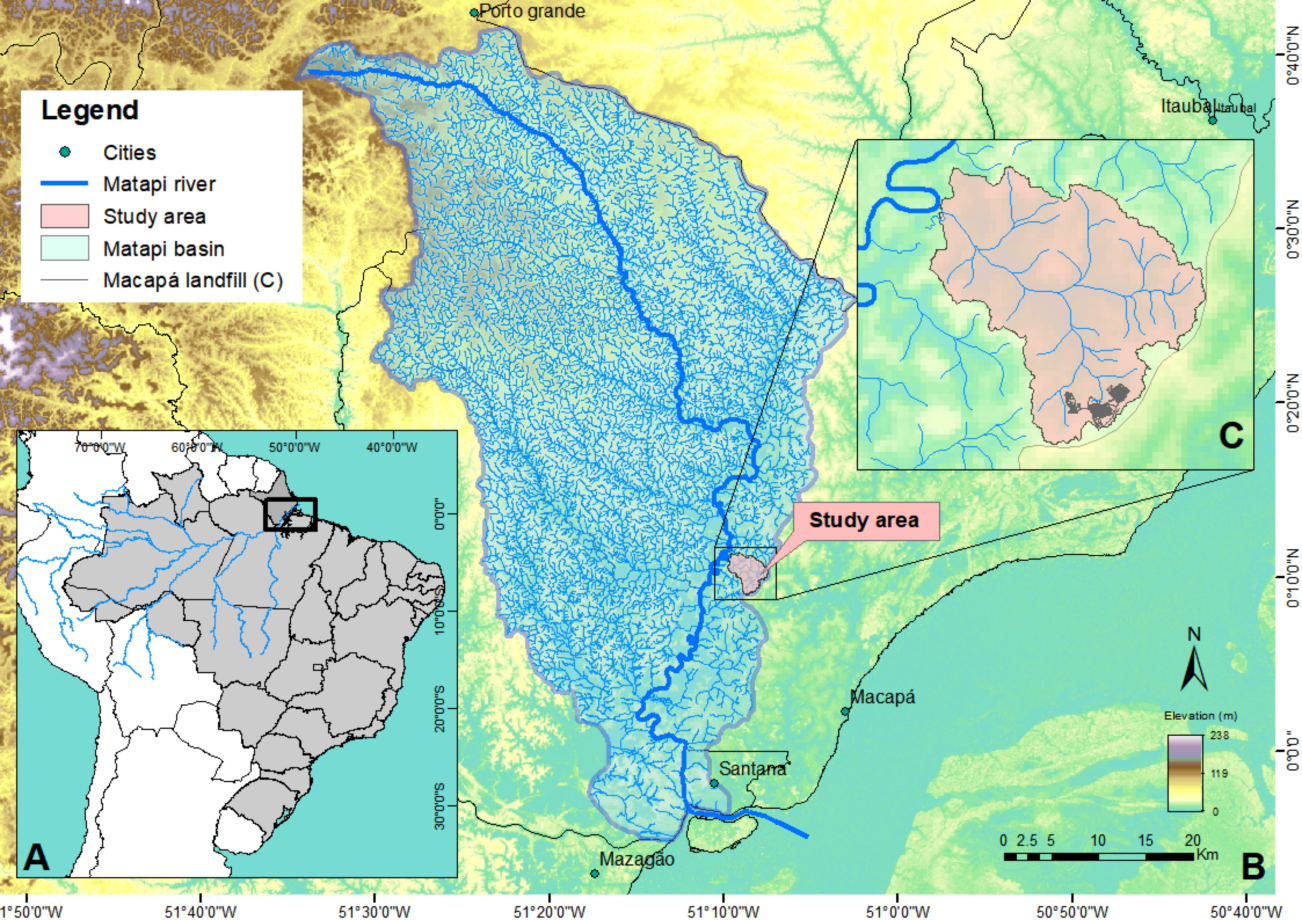
Study site location. (A) Geographic location in South America and Brazil, in the Amazonian estuary, (B) Matapi River Basin –Amapá, influenced by the tides; (C) specific study site and drainage sub-basin.

Matapi River basin is located in the coastal-estuarine sector of Amapá State –Brazil -, it covers Macapá, Santana and Porto Grande counties (Figs. 1A and 1B). Matapi River is tributary of Amazonas River, in its shallowest stretch. It is influenced by semidiurnal mesotides that often influence pollutants’ hydrodynamics and dispersion in the drainage system, and forest environments in flooded areas’ plains (Cunha et al., 2011). There is small human influence on this basin, although several human activities are evident in stretches close to its mouth: urbanization, livestock, agriculture, industrial district (Cunha et al., 2011; Cunha et al., 2021; Silva, Lima & Tavares-Dias, 2016; Neves & Tavares-Dias, 2019; Abreu et al., 2020; Souza et al., 2021; Felix et al., 2021) and ASMM’s expansion. The study site is inserted in Matapi River basin (0°10′19,63″N; 51°8′37,53″W), mainly in the drainage sub-basin (DSB), whose area is 11.08 km^2^. In geographic terms, the head of this DSB is located the ASMM (Fig. 1C), close to the left side of BR-156 road, 13 km from Macapá’s international Airport, and 3.5 km from the main channel in Matapi River (Leal, 2012).

The region’s topography presents limited natural precipitation drainage capacity, which is characteristic of very plain and winding rivers, at relatively low flow speed (Cunha et al., 2011; Cunha & Sternberg, 2018; Felix et al., 2021). On the short-term, it records speed of ≈1 m s^−1^ through 2/5 of the complete cycle of tides (12.9h). With respect to other periods-of-time, speed is approximately 0.5 m s^−1^. Because the study site is close to the equator line, the region has two well-defined climatic seasons: the rainy season (Jan–Jul), with mean precipitation Pm = 2,100 mm, and the dry season (Aug–Dec) (Pm = 178 mm) (Galardo et al., 2009; Kuhn et al., 2010).

**Figure 2:**
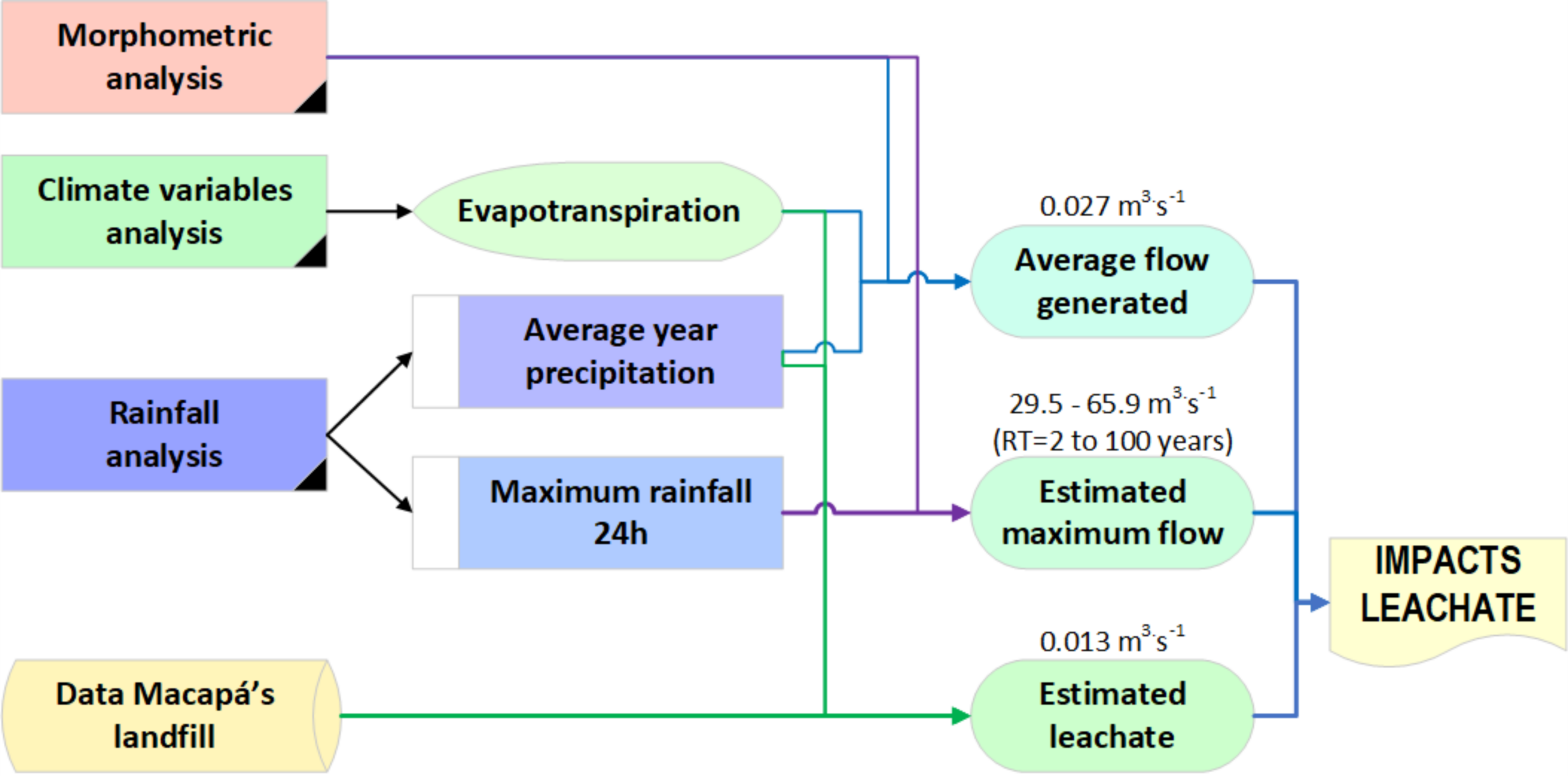
Flowchart of the main stages to estimate leachate in ASMM.

The study methodology followed the stages presented in the flowchart (Fig. 2):

### Generation of medium flow rates

This topic is about describing the flow generation process during normal weather events. That is, in a typical climatic year of average precipitation expected for DSB. Therefore, we describe the morphometric characterization, based on the analysis of climatic variables of precipitation and, eventually, temperature.

DSB was designed based on the Global Digital Elevation Model (GDEM) Aster 30 × 30. Its morphometric featuring was summarized as follows: (a) area (A) and perimeter (P), (b) form parameters, compactness coefficient (Cc), form factor (Ff), elongation ratio (Re), length and drainage index; (c) relief parameters related to terrain topographic variations, mean slope (j), mean height (Hm); and (d) linear measurements (Lb), drainage density (Dd) (Table 1).

**Table 1:** Morphometric parameters.

Morphometric parameters	Formula	References
Basin relief	*R* = *maxH* − *minH*, H (m)	Hadley & Schumm (1961); Strahler (1964)
Compactness coefficient	$Cc=0.282\ast \frac{P}{A} $ wherein A is area (km^2^)	Gravelius (1914); Campos Aranda (1998)
Form factor	$F= \frac{A}{{L}^{2}} $ wherein A is the area of the basin (km^2^) and Lb is basin length (km)	Horton (1932); Horton (1945)
Elongation ratio	$Re=1.128{A}^{ \frac{1}{2} }$/Lb wherein Lb is basin length (km)	Schumm (1956)
Mean basin elevation	*Hm* = Interpolation of each Partial area (a/A) and partial altitude (km2)	Curve hypsometric
Curve hypsometric	Partial area (a/A) with partial altitude (km)	Kouli et al. (2007)
Mean basin slope	*J* = 100∑*L*_*i*_*E*/*A* where L_i_= Sum of lengths of each contour (m), E = distance of contours (m)	Ibañez, Moreno & Gisbert (2011)
Drainage density	*Dd* = ∑*L*/*A* wherein L = total length of the stream (km)	Horton (1932)

**Figure 3:**
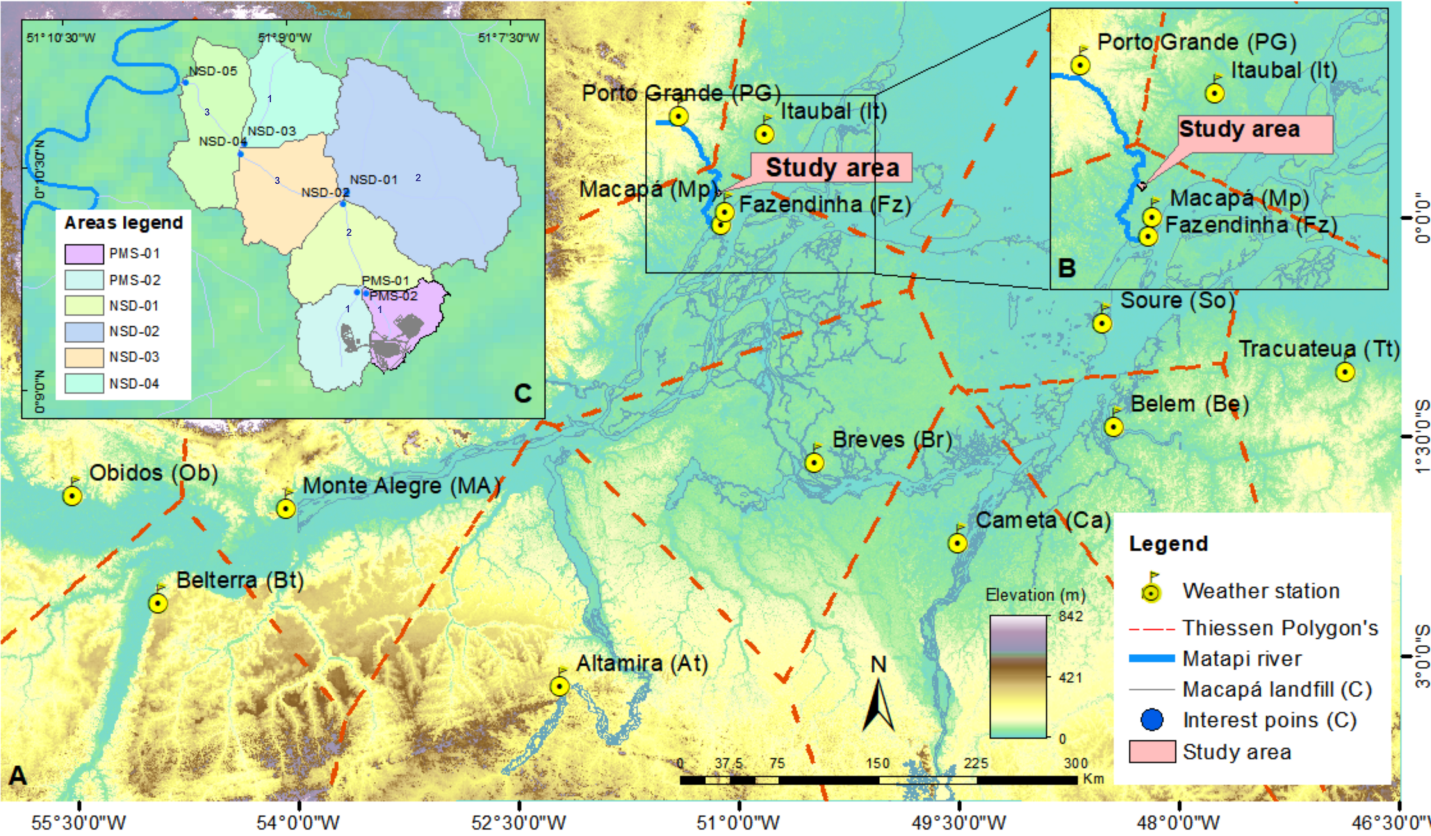
Meteorological stations and areas of interest. (A) Hydrometeorological stations used to determine extreme precipitation events, (B) meteorological stations to generate potential evapotranspiration, and (C) points of interest in the present research (PMS-01.PMS-02, NSD-01, NSD-02, NSD-03 and NSD-04) to estimate runoffs.

Climate variables such as mean temperature (Tx) and relative humidity (Ur) were calculated through regression test based on the data series of Fazendinha stations –Fz (0°3′S, 51°6′36″W, altitude 14.46 m), Macapá - Mp (0°2′6″N, 51°5′20″W, altitude 17 m) and Porto Grande - PG (041′40″N, 51°24′15″W, altitude 84 m) (1967–2018 data) (Instituto Nacional de Meteorologia, 2000) (Fig. 3B). Wv and Rs were found in the World Water and Climate Atlas of the International Water Management Institute (IWMI), at geographic coordinates 0°10′15″N, 51°8′37″W (centroid ASMM).

Hydrometeorological data were analyzed through the Penman Monteith method, which was modified by FAO (Allen et al., 2006) to generate ETo Eq. (1) in free software CROPWAT v8.0, as well as through processed information on the meteorological variables Fig. S1 and Table S1).

(1) \begin{eqnarray*}ETo=c \left[ w\mathrm{ \ast }Rn+ \left( 1-w \right) \mathrm{ \ast } \left( f \left( u \right) \mathrm{ \ast } \left( ea \left( 1- \frac{Ur\text{%}}{100} \right) \right) \right) \right] \end{eqnarray*}

where *ETo* = Potential Daily Evapotranspiration (mm day^−1^); *c* = Penman adjustment factor; *w* = Penman’s weighing factor; *Rn* = total liquid radiation (mm day^−1^); *ea* = water steam pressure at saturation (mbar); *Ur* = Relative Humidity; $f(u)=(0.25\ast (1+ \frac{Wv}{100} ))$ = wind function; *Wv* = mean wind speed measured 2.0 m from the ground (km dia^−1^).

The time series used for hydrometeorological monitoring were obtained from the stations of Fz, Mp, PG (INMET - longest and faulty series) and ASMM (shortest and current series) over the time interval between January 1, 1967, until December 31, 2018. However, possible series inconsistencies may cause significant errors. This fact occurs when gaps in the identification or elimination of data arise (Tarazona, 2005).

The graphic method was adopted to plot hydrograms (Fig. S2) that are visually simpler to show the outcomes (World Meteorological Organization (WMO), 2011; Liu, Wang & Liu, 2017; Moreira et al., 2020), as well as to detect and identify likely drastic changes in the historical series and/or in their trends. Accordingly, it was possible to estimates the periods-of-time whose intervals are scarce (Autori dad Nacional del Agua, ANA; Lema, Plaza & Cecilia, 2019) or reliable (Serrano et al., 2012; Shi et al., 2019).

Student’s *t*-test assessed whether the different precipitation measurements in the selected periods-of-time were significant (*p*-value <0.05) by determining likely drastic changes. The F-Fisher test assessed whether the selected periods-of-time presented standard deviations significantly similar or different (*p*-value <0.05) Eq. (2). Whenever outcomes presented significant differences in the series (Table S2), they were adjusted through Eq. (3) (Villón, 2016), in order to correct the information (Table S3) based on the following statistical impositions:

\begin{eqnarray*} \left\vert Tc \right\vert \leq \left\vert Tt \right\vert \left( 95\text{%} \right) \Rightarrow \overline{{x}_{1}}=\overline{{x}_{2}}(\text{means are similar}) \end{eqnarray*}

\begin{eqnarray*} \left\vert Tc \right\vert \gt \left\vert Tt \right\vert \left( 95\text{%} \right) \Rightarrow \overline{{x}_{1}}\not = \overline{{x}_{2}}(\text{different means or there are changes}) \end{eqnarray*}

\begin{eqnarray*}Fc\leq Ft \left( 95\text{%} \right) \Rightarrow {S}_{1}(x)={S}_{2}(x)(\text{standard deviations are statistically similar}) \end{eqnarray*}

(2) \begin{eqnarray*}Fc\gt Ft \left( 95\text{%} \right) \Rightarrow {S}_{1}(x)\not = {S}_{2}(x)(\text{standard deviations are statistically different})\end{eqnarray*}

(3) \begin{eqnarray*}{x}_{ \left( t \right) }= \frac{{x}_{t}-\overline{{x}_{1}}}{{S}_{1}} .{S}_{2}+\overline{{x}_{2}}\end{eqnarray*}

where *x*_(t)_ is the corrected value, *x*_*t*_ is the value to be corrected, $\overline{{x}_{1}}$ is the average of data from the doubtful period, *S*_1_ is the standard deviation of data recorded for the doubtful period, $\overline{{x}_{2}}$ is the average of data in the reliable period, and *S*_2_ is the standard deviation of data from the reliable period.

Lack of data shortage in estimated precipitation records obtained through the models were completed by extending data of pre-existing series. Similar analysis was carried out by using several methods (linear regression, multiple regression, means of neighborhoods, mean ratios, correction in comparison to neighbor stations) (National Meteorology and Hydrology Service of Peru (SENAMHI), 2013). The linear regression method consists in establishing a linear regression and correlation between a consistent station and another one that lacks information (Varela, 2007). In order to fulfil and extend the stations (Table S4), they were grouped based on their proximity and similarity to altitude and climate, in case the existence of more representative and reliable stations was confirmed. Accordingly, the series were statistically assessed through the Student’s *t* and F-Fisher tests, with emphasis on the analysis method known as “double mass” (SearcLUy, Hardison & Langbein, 1960). Correlation, in this method, is found by plotting the value of mean accumulated annual precipitation per station on the abscissa axis, and the accumulated annual precipitation value on the ordinate axis (Fig. S3). The selection of the most reliable model was carried out based on the smallest number of series’ discontinuity in order to generate precipitation per station (Varela, 2007; National Meteorology and Hydrology Service of Peru (SENAMHI), 2013) (Figs. S4 and S5).

Values recorded in the ASMM were taken into consideration to feature precipitation given its effective proximity and representativeness in Thiessen method application. Ratios were altitude similarity, coverage and relief. These stations allowed using the hydrological Lutz Scholz model (Lutz Scholz, 1980), which was defined as deterministic (hydric balance) to calculate monthly runoff for an average year. The Lutz Scholz model was chosen because it allows combining factors that produce and influence generated mean runoffs (Qg) (Sarazú Cotrina, 2018).

The main parameters used in the model were precipitation (Q_p_), evaporation (Q_ev_), storage (Q_ar_) and the natural exhaustion function (Eq. 4). The stochastic mathematical model (Markovian process) was used to calculate Q_g_ (Eq. 5). Subsequently, the statistical analysis and data correction were carried out through Eq. (3).

(4) \begin{eqnarray*}a=0.00252\ast Ln \left( A \right) +0.026\end{eqnarray*}

where *a* is the exhaustion coefficient per day, *A* is the DSB area (km^2^).

This equation enables estimating exhaustion, which was considered median, because it presented retention close to 80 mm year^−1^, as well as mixed vegetation (grass, woods and cultivated terrains) (Aguirre, 2006).

(5) \begin{eqnarray*}{Q}_{g}={b}_{1}+{b}_{2}{Q}_{t-1}+{b}_{3}P{E}_{t}+Z.E.\sqrt{1-{r}^{2}}\end{eqnarray*}

where *Q*_*g*_ is runoff in month t; *b*_1_, *b*_2_, *b*_3_ are multiple linear regression coefficients, *Q*_*t*−1_ are runoff in the previous month, *PE*_*t*_ is the effective precipitation in month t, *Z* is the often distributed random number (0.1) in month t, *E* is the standard error of the multiple regression, and *r* is the multiple correlation coefficient.

Besides generating the Q_g_ of the study site, additional Q_g_ were also generated based on area of interest (PMS-01, PMS-02, NSD-01, NSD-02, NSD-03, NSD-04, NSD-05) (Fig. 3C). The PMS-01 is the critical area where the ASMM is located. The criterion for choosing these areas was based on the existence and distribution of small river channels that connect to the mainstream of the DSB. DSB can be defined as exoreic–pluvial classified in the fluvial hierarchy category as third order.

### Estimated maximum flow

An information correction process was carried out by multiplying the maximum historical precipitation value within 24 h for each station identified by Fc = 1.13 (Factor of reading correlation, recommended by WMO).

*Pmx24h* records were analyzed with the Hydrognomon software v.4.01, which allows determining up to 24 distributions per station. This process aimed at assessing the (D_max_) adjustment through the Kolmogorov–Smirnov test applied to each distribution (Normal, Log-Normal, Exponential, Gamma, Person III, Log-Person III, Gumbel Max, EV2-Max, Gumbel Min, Weibull, GEV Max, GEV Min, Pareto, Normal L-Moments, Exponential L-Moments, EV1-Max L-Moments, EV2-Máx L-Moments, EV1-Min L-Moments, EV3-Min L-Moments, GEV Max L-Moments, GEV Min L-Moments, Pareto L-Moments L-Moments).

The main aims were to choose the best distribution with the greatest significance (D_max_) at *p* < 0.05 (Kozanis et al., 2010; Güçlü, Şişman & Yeleğen, 2018) and to calculate *Pmx24h* in short return times (RT): 2, 5, 10, 25, 50 (Marazuela et al., 2019), as well as in longer times, such as the 100 one (Masseran & Safari, 2020). RTs were generated in the Hydrognomon software. The calculated values were interpolated through the Kriging method (Ghosh, Pal & Santra, 2020), with the aid of ArcGIS, to originate the isohyets.

The produced isohyets are curves that connect equal-precipitation points (SearcLUy, Hardison & Langbein, 1960; Herrmann & Bucksch, 2014). As an indirect method, it consists in estimating the *Pmx24h* in the area of the likely maximum precipitation event in the basin (WMO, 2011). The isohyets were generated from the RT of *Pmx24h* (2, 5, 10, 25, 50 and 100 years) by taking into account all stations (Marazuela et al., 2019).

*Pmx24h* of DSB was estimated based on Eq. (6), for each RT. For instance, *Pmx24h* was found based on the weighed mean relation of the area between isohyet curves and isohyets’ values (Chow, Maidment & Mays, 1994). The rational method (Eq. 7) was adopted to estimate the maximum runoffs (Q_max_) (Villón, 2016; Colorado Department of Transportation, 2019).

(6) \begin{eqnarray*}P= \frac{{P}_{1}\ast {A}_{1}+{P}_{2}\ast {A}_{2}+\ldots +{P}_{n}\ast {A}_{n}}{{A}_{\mathrm{total}}} \mathrm{or} P=\sum \frac{Ai\times Pi}{{A}_{Total}} \end{eqnarray*}

where P is *Pmx24h* of the component (mm), *A*_*i*_ isthe area between each isohyet curve (km^2^), *A*_*total*_ is the total area of the DSB (km^2^) and *P*_*i*_ is the mean precipitation between the isohyets (mm)

(7) \begin{eqnarray*}{\mathrm{Q}}_{\mathrm{g}max}= \frac{CIA}{3.6} \end{eqnarray*}

where Q_gmax_ is the maximum estimated runoff (m^3^ s^−1^), *C* is the runoff coefficient –which depends on the vegetal coverage (American Society of Civil Engineers, 2008; World Meteorological Organization (WMO), 2011), on slope and soil type (dimensionless), *I* is the main maximum intensity –for duration equal to the concentration time –and the given return time (mm h^−1^) (Ministry of Transport and Communications of Peru, 2011; World Meteorological Organization (WMO), 2011); *A* is DSB area (km^2^).

### Leaching flow estimates

Predictions from leachate runoff in the projected controlled landfill, which took place in two different ways. The Swiss (Eq. 8) and rational (Eq. 9) methods were applied to estimate leachate production; these methods were substantiated by analyzed basic hydrological parameters (*Pm* and potential evaporation, data resulting from the application of Lutz Scholz mathematical model). After this stage was over, landfill estimates were summed (for each method) in order to determine leachate runoff, since it would represent the quantitative of the three Accumulation Ponds (AP) in ASMM. Terrain drains headed towards these APs.

(8) \begin{eqnarray*}Q= \frac{1}{t} P.A.K\end{eqnarray*}

where Q = mean slurry or leachate flow (L s^−1^); P = mean annual precipitation (mm); A = landfill surface area –ASMM (m^2^); t = time in seconds over the year (31,536,000 s year^−1^); K = coefficient based on the degree of waste compactness (poorly compacted landfills with specific weight of 0.4−0.7 t m^−3^) –estimates show that leachate production ranges from 25% to 50% (*K* = 0.25 –0.50), which concerns mean annual precipitation and corresponds to the landfill area.

(9) \begin{eqnarray*}{Q}_{m}= \frac{{P}_{m}-ES-EP}{t} A\end{eqnarray*}

where Q_m_ = mean generated slurry flow (m^3^ month^−1^), P_m_ = mean annual precipitation (mm), EP = mean evapotranspiration (mm); A = contribution area - ASMM (m^2^); t = time in seconds over the year (31,536,000 s year^−1^); ES = (P_m_ x C) = surface runoff (mm); C = surface runoff coefficient (dimensionless runoff).

Predictions helped comparatively analyzing the mean runoffs calculated for different interest areas (PMS-01, PMS-02, NSD-01, NSD-02, NSD-03, NSD-04, NSD-05) (Fig. 3C) and the maximum rainy periods that were projected in different RTs.

Furthermore, considering different RTs for ASMM, the maximum daily leachate production was estimated using Pmx24 h precipitation as an independent variable. In turn, Pmx24 h was calculated from Eq. (8). But it is important highlighting that estimates are necessary because runoff values are currently unknown due to multiple changes and adaptations, such as the expansion of project cells.

## Results

### Generation of medium flow rates

**Figure 4:**
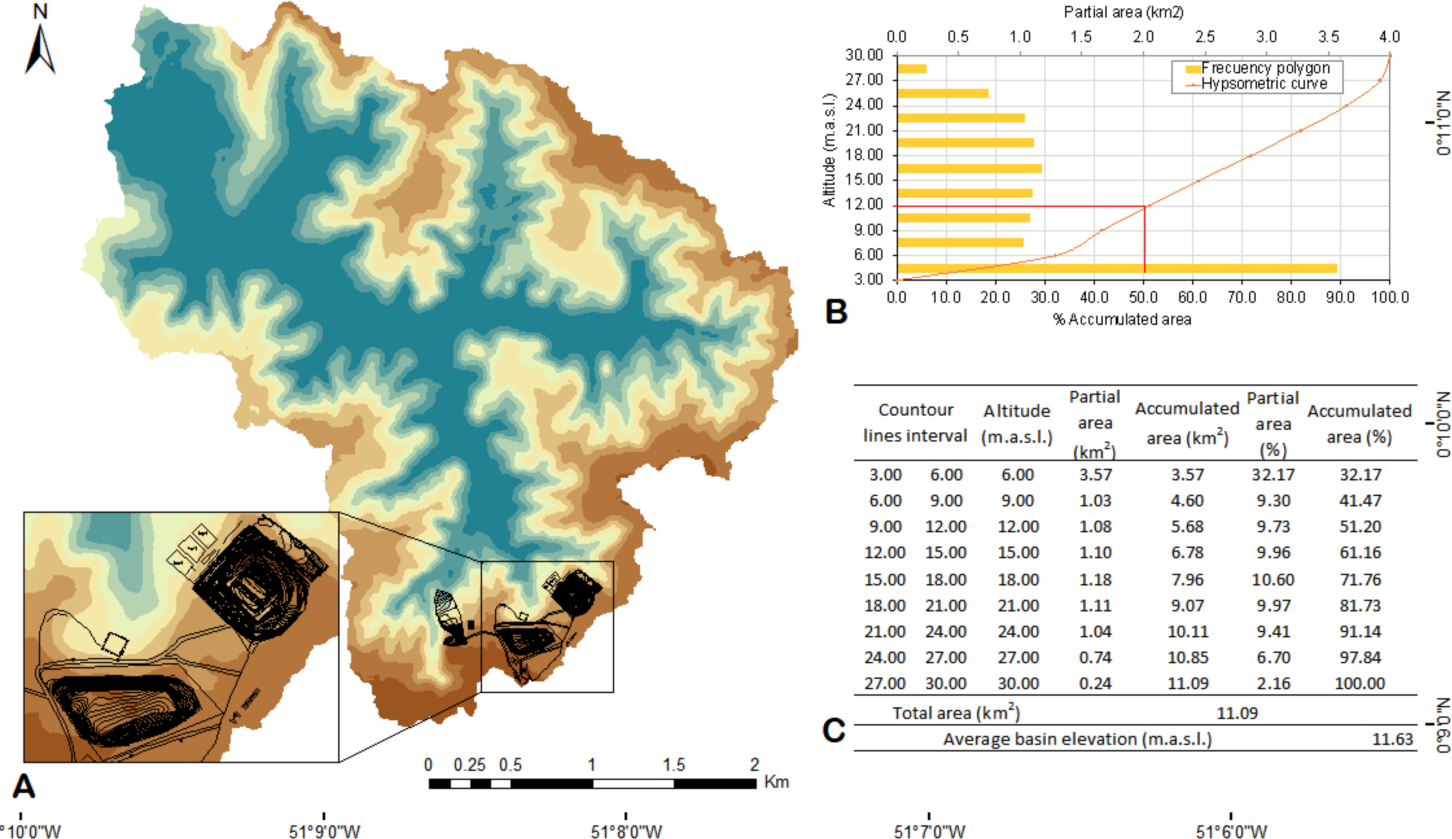
Hypsometric curves. (A) DSB’s hypsometric curves, ASMM highlighted in black; (B) frequency of altitudes in relation to the areas; (C) values of hypsometric curves’ level (mean elevation calculation).

Based on the Cc = 1.60 calculation, DSB is small and classified as Class III (Oval-oblong to rectangular-oblong) (Viramontes Olivas et al., 2018). It is slightly long (Ff = 0.32) (Delgadillo & Moreno, 2012) and presents low relief (Re = 0.83). The basin’s mean height (H_m_) is 11.63 m, and the isometric curves (Fig. 4) are estimates based on the deposition and erosion processes (Hurtrez, Sol & Lucazeau, 1999; Asfaw & Workineh, 2019). In this case, C_l_ = 5.92 km defines the largest mean extension throughout a straight line between the exit and the water parting. The longitudinal extension of the main channel (E_l_ = 4.54 km) is defined as the longest effective distance naturally crossed by the water flow in the DSB (Table S5). DSB’s Dd = 0.53 is classified as low density (Delgadillo & Moreno, 2012) and this finding points towards highly resistant strata, dense vegetation and low relief. High D_d_ values are indicative of weak or impermeable rocks under sparse vegetation and hilly relief (Strahler, 1964; Aladejana & Fagbohun, 2019) (Table S5).

Q_g_ showed significant variation between monthly means for an average year (*p* < 0.05) (Table 2). Values 0.071 and 0.723 m^3^s^−1^, respectively, represent the runoff in October (minimum) and April (maximum). Similarly, such a process took place in the interest points, PMS-01, PMS-02, NSD-01, NSD-02, NSD-03, NSD-04, NSD-05 (Table 2). Estimates for Q_g_ differences enabled calculating the concentrations of chemical elements deriving from different sources (soil, vegetation, industrial activity—as observed in the landfill) that interact with the medium (Barros, 2013).

### Maximum flow estimate

**Figure 5:**
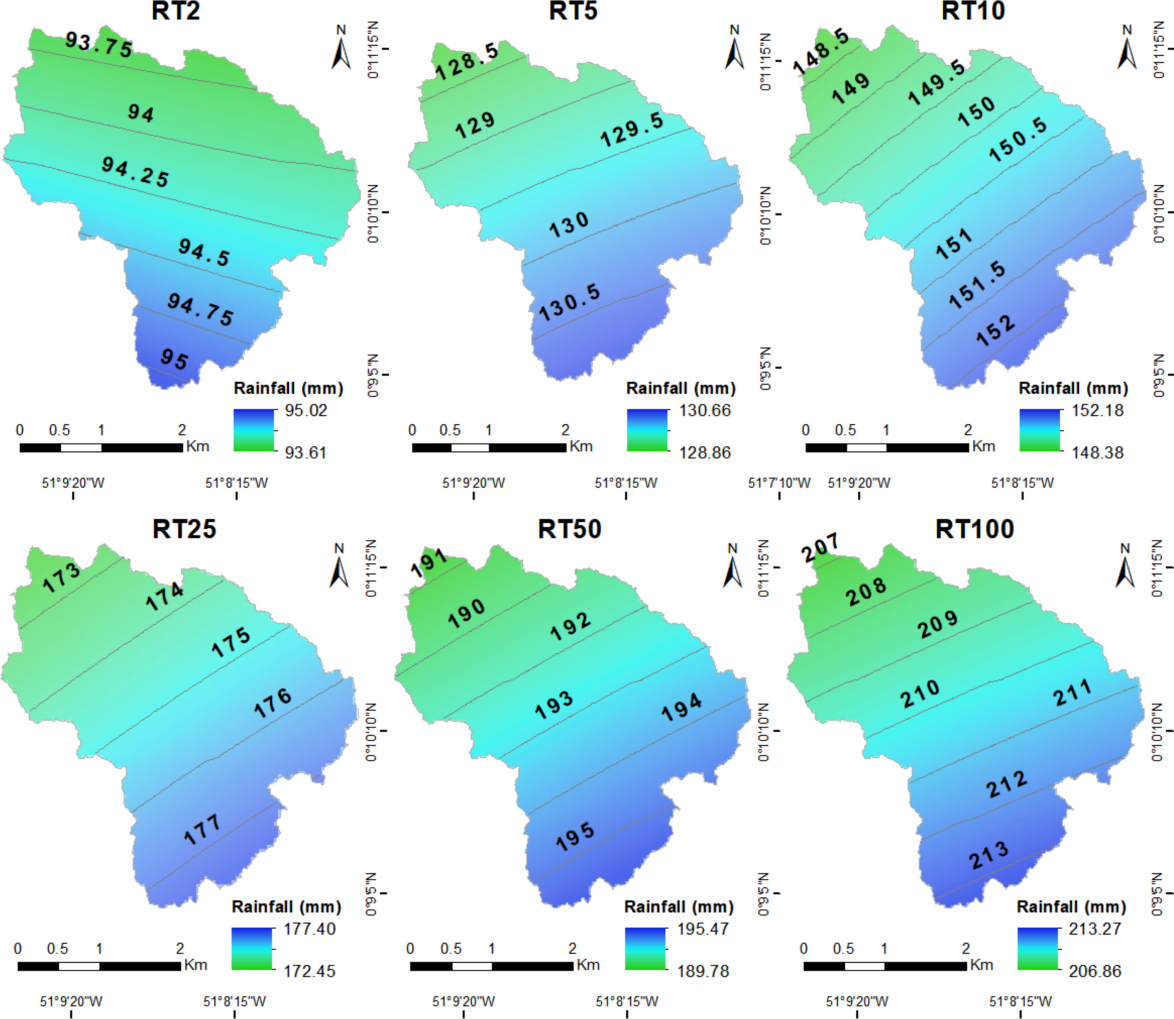
Comparison of estimated maximum rain distribution in different RTs.

Table S6 presents the best distribution models and adjustment data (D_max_) per station (Fig. 3A). These models allowed calculating *Pmx24h*, which were conditioned by RT (2, 5, 10, 25, 50 and 100 years). The isohyet curves produced (Fig. 5) for each RT were the final products of the subsequent predictive distribution (Villalta, Bravo deGuenni & Sajo-Castelli, 2020).

**Table 2:** Mean monthly generated flows (m^3^ s^−1^) (Qg).

Description	Jan	Feb	Mar	Apr	May	Jun	Jul	Aug	Sep	Oct	Nov	Dec
	Qg (m^3^ s^−1^)											
Study area	0.599	0.704	0.722	0.723	0.660	0.583	0.486	0.276	0.115	0.071	0.119	0.327
PMS-01	0.041	0.047	0.046	0.045	0.038	0.033	0.028	0.014	0.006	0.004	0.004	0.018
PMS-02	0.055	0.064	0.061	0.060	0.052	0.044	0.037	0.019	0.008	0.006	0.007	0.025
NSD-01	0.171	0.197	0.200	0.199	0.152	0.138	0.119	0.060	0.026	0.017	0.018	0.071
NSD-02	0.212	0.244	0.252	0.252	0.187	0.173	0.149	0.081	0.034	0.021	0.022	0.088
NSD-03	0.460	0.528	0.545	0.546	0.416	0.374	0.321	0.182	0.083	0.054	0.059	0.194
NSD-04	0.061	0.070	0.073	0.075	0.052	0.050	0.044	0.027	0.013	0.007	0.008	0.025
NSD-05	0.599	0.687	0.723	0.721	0.536	0.489	0.424	0.249	0.112	0.056	0.074	0.242

Figure 5 represents comparative isohyets of precipitation projected based on the Kriging method in Different RTs. Simulated rain occurrence results with closer values headed Northeast-Southeast (RT = 2 years), and it contrast the other periods from Northeast to Southeast. There are variations in the precipitation map ranging from 94.2 mm (RT = 2 years) to 210.2 mm (RT = 100 years) (Table 3). Variation in isohyets’ direction happens due to changes in wind direction and to increase in precipitation (Pearson III distribution, Table S6, Mp station).

**Table 3:** Estimated maximum flows.

RT	P_max design_	P_d_	I	Q_gmáx_
(years)	(mm)	(mm)	(mm h^−1^)	(m^3^s^−1^)
2	94.2	46.8	31.9	29.5
5	129.6	64.4	43.9	40.6
10	150.5	74.8	51.0	47.1
25	175.2	87.1	59.4	54.9
50	192.8	95.9	65.4	60.4
100	210.2	104.5	71.3	65.9

**Notes.**

Rain-duration relation (P_d_), rain intensity (I), Pmax design for 24 h.

Q_gmax_ recorded 0.30 of runoff coefficient, and delay coefficient reached 0.60; these values were compatible with basins presenting deciduous forests (Chin, 2013; LMNO Engenharia, Pesquisae Software, 2014) and deciduous foliage (Colorado Department of Transportation, 2019). Total precipitation within one hour (P_d_) increased from 46.8 to 104.5 mm and it accounted for increasing Q_gmax_ from 29.5 to 65.9 m^3^ s^−1^, for different RTs (Table 3).

### Leachate flow rates

**Figure 6:**
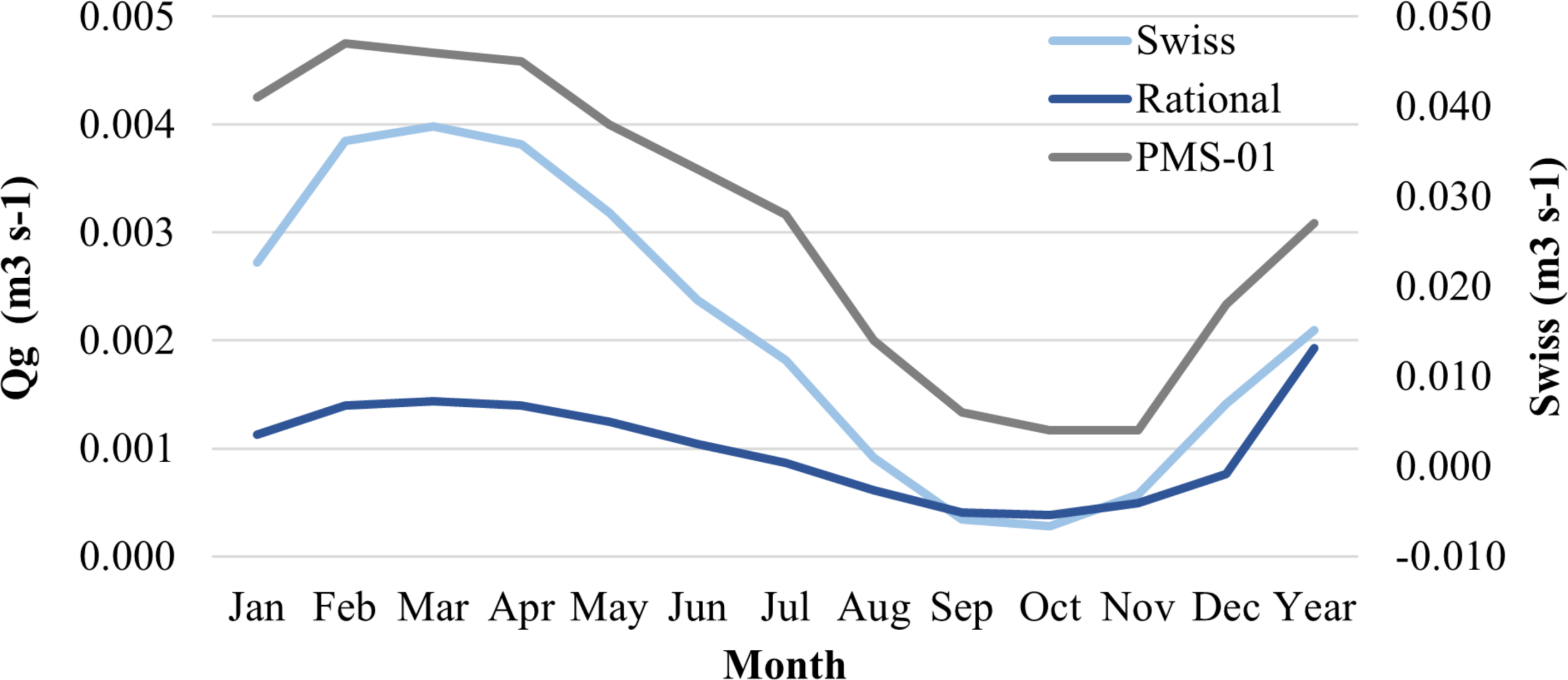
Comparison between generated runoff and leachate from an average year.

Leachate runoff generation based on the Swiss and rational methods allowed observing significant variation between monthly means recorded for one typical average year (*p* < 0.05). For instance, values based on both the Swiss (0.0003 and 0.0040 m^3^ s^−1^) and rational methods (−0.0055 and 0.0072 m^3^ s^−1^) well-represent the generated leachate runoff, mainly in months presenting extreme precipitation—March (maximum) and October (minimum)—since it indicates the lowest values in August and December. On the other hand, values got concentrated below the generated runoff in the interest area (PMS-01), which is highlighted in grey (Fig. 6 and Table S7).

We observed that the variations in the maximum leachate flow estimated by the ASMM in 24 h vary between 28.8 m^3^s^−1^ (RT = 2 years) and 64.6 m^3^s^−1^ (RT = 100 years). These values are concentrated close to the maximum flow estimated for DSB between 97.5% (RT = 2 years) and 98.1% (RT = 100 years) (Table 4). It is important to note that Q_gmax_ was calculated disregarding the interference of leachate production.

**Table 4:** Maximum leachate flow estimated in 24 h.

**RT (years)**	Q_gmáx_ - DBS (m^3^ s^−1^)	**Leachate (m**^3^ s^−1^)	Ratio^*^ **(%)**
2	29,5	28,8	97,5
5	40,6	39,6	97,5
10	47,1	46,1	97,9
25	54,9	53,7	97,8
50	60,4	59,2	98,0
100	65,9	64,6	98,1

**Notes.**

* The estimated leachate flow ratio based on estimated Qgmax of DBS.

## Discussion

The investigation of morphometric parameters was useful to identify runoff scenarios with the aid of the SIG tool. The estimated flows represent a region with relative rainfall data shortage; therefore, after the analytical syntheses, the morphometric description allowed identifying the low drainage density (0.53 km^−2^) due to the average topography relief, as it enables determining latitude correlation (6%). This information provides useful information about the hydrological features for future projects (or research) that need such hydrological parameters. When it comes to more robust studies, the previously described parameters had to be combined to geological and chemical variables that remain relatively scarce for scales necessary for landfill projects in the region. However, because the hydrometeorological response was found at reduced scale (DSB level in Matapi River), temperature increase (0.5 °C to 1.9 °C, in summer and 0.3 to 1.2 °C, in winter) throughout the assessed period seem to diverge from the PBMC prediction (2012) to the entire Amazon (1 °C to 5 °C, in summer and 1.5 °C to 6 °C, in winter) along the current century (Cunha et al., 2014). The recorded precipitation maximums were above 400 mm in summer and above 600 mm in winter. Oliveira & Cunha (2014) recorded values higher than 1,000 mm in summer in Amapá State, by the coastal shore. This period is influenced by ITCZ (Intertropical Convergence Zone), whose central position in the tropical cloud band is located in equatorial latitudes during these months. Therefore, precipitation presented changes in trend in comparison to that in the study by Moreira et al. (2020) and; Moreira, Cunha & Costa (2021), which did not present statistical significance. This finding may have resulted from the fact that the analysis was used as a total, and as short period in the present research.

Q_g_ found through the Lutz Scholz model (Table 2) was calculated because DSB is easy to access and is smaller. The model’s correlation coefficient was 99%, and it was comparatively equal to, or higher than, that of similar studies that have recorded values up to 98% (Tarazona, 2005), 92% (Gamarra, 2018), 96% (Pineda, 2015), 99% (Tito Betancur, 2018). However, the Lutz Scholz model presented some limitations, such as specific use to calculate monthly runoff. For instance, there are several gaps and challenges concerning the application of this model. In other words, nowadays, specific studies carried out in tropical forests in South America were not found in the literature; hence, its application has been used in mountain and coastal regions so far (Tarazona, 2005; Pineda, 2015; Gamarra, 2018; Tito Betancur, 2018).

High Q_gmax_ can actually result from extreme precipitation events (>209.97 mm, TR ≥100 years) (Demirdjian, Zhou & Huffman, 2018; Li et al., 2020); therefore, it is important taking into consideration the simulations presented in Fig. 5 and the deriving maximum runoffs (Table 3). These maximum runoffs are oftentimes linked to environmental and public health impacts on the surrounding areas (landslides, floods, pathological illnesses, among others): they affect the close locations. Therefore, it is important monitoring the physical-chemical parameters and their concentrations, which are bond to the discharge from the main activities in the sanitary landfill, as these parameters interact with water. However, nowadays, this information is not available (Naveen & Malik, 2019; Sharma, Ganguly & Kumar Gupta, 2020).

P_d_ increase provides runoff increase after the intensity-duration-rain frequency approach (IDF). Runoff increase is controlled through critical precipitation duration achievement because of Pd increase. Consequently, Q_gmax_ can decrease due to rain intensity (Baiamonte & Singh, 2017). The advantage of it lies on the fact that Q_gmaxis_ also extremely useful to estimate potential water surpluses in the study site or to substantiate other project parameters that depend on similar climatic factors (Sousa, Cunha & Cunha, 2021).

It was observed that the estimated leachate in LA was below the runoff generated for the area of interest (PMS-01, area where ASMM is located in). In this case, they represented ≈7.76% (Swiss method) and 48.74% (rational method), on average, of the runoff in PMS-01. In case of extreme events, it was also not possible highlighting the likelihood of having leachate overflow in ASMM. Such a probability represents considerable hydrological-sanitary risk, as there are no protection mechanisms to these cases. Furthermore, although ASMM has a leachate recirculation system, there are considerable gaps in knowledge about the occasional overflow of this leachate—such events can be occurring in landfilled cells. Talalaj & Biedka (2015) have stated that recirculation can increase leachate concentration with sulfate and other compounds, depending on the pre-treatment performed in the stabilization ponds. With respect to leachate drainage system in Macapá, theoretically, there are significant differences in leachate production when different drainage systems are used (Flores, Cunha & Cunha, 2021).

Yet, it is important highlighting that the rational method allowed estimating one single flow “reversion” in August and December. Evaporation and temperature in these months were higher, although there was lesser precipitation. It is worth pointing out that vegetation (smaller plants) in ASMM may be a factor accounting for changing leachate production. For example, some plant species, in combination to root depth, can represent a contention effect of considerable precipitated water on leachate generation. Tree planting tends to decrease the mean annual leachate generation rate by ≈15% (Tozetto, 2008).

The current study has shown that rain intensity enables assuming that part of the precipitation variability could significantly affect water in DSB and leachate flow in the sanitary landfill during local maximum flow peaks, because the highest *Pmx24h* values in different RTs tend to take place right close to ASMM (Fig. 5). In case there is an extreme precipitation event, consequently, there would be the probability or proportionality of runoff elevation in DSB (Q_gmax_) –it would range from 29.5 m^3^s^−1^ (RT = 2 years) to 65.9 m^3^s^−1^ (RT = 100 years). In this case, the existence of the sanitary landfill was not considered.

On the other hand, leachate production would also be affected by precipitation (Fig. 5) reaching values between 28.8 m^3^s^−1^ (RT = 2 years) and 64.6 m^3^s^−1^ (RT = 100 years). That is, proportionally, it would result in a ratio between 97.5% and 98.1% of DSB, whose impacts would be significant, generating possible overflows in DSB, because the transport of chemical compounds (leachate or slurry) would proportionally increase due to flow discharge (Bergstrom et al., 2016). These processes can lead to peaks capable of easily exceeding the transport capacity in the planed and projected rainfall drainage networks, a fact that could also happen in any similar sanitary landfill (Pagliara et al., 1998; Barros, 2013).

Biochemical parameters that present lower rainfall concentration values in the drought season and higher values in the rainy season were recorded in Southern Amapá State (Oliveira & Cunha, 2014). Therefore, in case there are extreme rainfall events in the Southern part of DSB (ASMM location zone), the epidemiological risks would be greater given the higher potential for water surplus in the basin or overflows in accumulation ponds (Sousa, Cunha & Cunha, 2021). Water speed could increase during relatively shorter periods-of-time and they could reach approximately 1 m s^−1^, which could eventually match the maximum peaks of the syzygy tides by ≈1/2 of its complete semidiurnal cycle (≈12.9h), although this area presents a low level of drainage (Cunha et al., 2011).

In this case, there is a consensus in the literature on what are the impacts caused by climatic and anthropogenic factors that influence the seasonal patterns of leachate flow in landfills (Chai et al., 2020). Such climatic factors already appear to be impacting the short- and long-term hydrological pattern in the Matapi River (Felix et al., 2021). Consequently, these impacts will likely be propagated towards tributaries relatively close to the Macapá sanitary landfill.

Leachate produced in ASMM can reach considerable volume and load in the waterbodies due to its assumed interaction with the soil in the basin—the soil is hydro-geo-chemically affected in aquatic medium. In case of leachate concentration peaks and eventual influence on the cell discharge, the probability of overflow in the ASMM would increase. This process would probably extend to the Matapi River. In these cases, riverside communities and surrounding aquatic ecosystems would be environmentally impacted (Cunha et al., 2021), mainly fishermen and the aquaculture sector (Costa et al., 2022).

The performed estimates enable further studies to determine how leachate influenced by precipitation can have impact on locations and on the health of the population living in the surrounding areas, because leachate in sanitary landfills is an important source of per- and polyfluoroalkyl substances (PFAS) in the environment. PFAS may account for severe impact on ecosystems and on human health (for example, it can cause cancer, weaken the immunological system and stop thyroid hormone) (Feng, Song & Mo, 2021). The evaluation of groundwater and surface water quality can slowly decrease around the landfill given the emergence of sanitary landfill leachate, because these values usually exceed the limit prescribed by WHO (Mishra et al., 2019).

If there are structural changes, such as those that recently took place in the configuration of cells and on their coverage in ASMM, and if the hydrological effects are ignored, mainly in areas where precipitation peaks are concentrated, it is possible to have errors by ≈100% in rates predicted for annual leachate generation projects. It mostly happens because of scarce data availability in the field. Accordingly, it is recommended to conduct new studies in order to propose models that include vegetation influence on leachate production. It is important to note that the new legal framework for Brazilian sanitation (Law N°14.026: Brazil, 2020) represents a brutal failure in basic sanitation policy, where the focus is essentially on large urban centers. Therefore, it disregards the urban and rural needs of most small municipalities in the Amazon. This criticism can be seen both for urban and rural areas scattered in the Amazon, as they are generally lacking in information and reliable parameters to develop customized projects for the solid waste sector (Flores, Cunha & Cunha, 2021).

## Conclusions

The adoption of the Lutz Scholz hydrological model allowed the locating of the mean surface runoff of 0.452 m^3^ s^−1^, and minimum mean value of 0.071 m^3^ s^−1^ (October), as well as maximum mean value of 0.723 m^3^ s^−1^ (February); the correlation coefficient was R = 99%, without significant differences in trend changes. The most representative runoff generations were PMS-02 and PMS-01, with runoffs of 0.0374 m^3^ s^−1^ and 0.0371 m^3^ s^−1^ per km^2^, respectively, depending on the location and on its corresponding areas for final discharge obtainment.

Based on the estimates, the main runoff area contributing to discharge in Matapi River is located close to ASMM (PMS-01), and it was shown that this is a place of relevant risk concerning hydro-sanitary events. Therefore, the detailed balances of leachate, and of the other flows, during LF implementation project should be deepened. On the other hand, the estimates used for leachate allow us to have a perspective of the possible effect of precipitation on the DSB in extreme situations. Although it is not compared with real data of the production of leachate in the landfill.

The present study has also shown that rain intensity allows assuming that part of its variability significantly affects water and leachate flow within cells of the sanitary landfill, mainly during peak events (maximum flows). Furthermore, in case of extreme precipitation events, there would consequently be proportional elevation in runoffs, which could range from 29.5 m^3^ s^−1^ (RT = 2 years) to 65.9 m^3^ s^−1^ (RT = 100 years) at the sub-basin between 28.8 m^3^s^−1^ (TR = 2 years) and 64,6 m^3^s^−1^ (TR = 100 years) at the landfill. In this case, the models were very useful to estimate expected typical and maximum runoffs even in situations of little influence of precipitation. This fact accounted for improving the perception about the dynamics of the drainage system in the relevant area of Matapi River basin (≈97,8 ± 0.3, ASMM∼ DSB). Additionally, ASMM estimated leachate was lower (48.15%) than the generated runoff in the current research, in an average year, in the PMS-01 area.

The models used in the current study can be integrated to other geo-chemical and geological studies aimed at assessing potential impacts of sanitary landfills and on their surrounding areas, in Eastern Amazon. Consequently, the present research has shown that the potential risks of leachate leakage are real. However, these risks seem to stem much more from the low level of monitoring and management of the water balance or leachate than from natural weather events, especially in cases of extreme events. Therefore, further technical studies would be necessary to assess the environmental sustainability of the sanitary landfill in the short, medium and long term, so that these risks observed during the leachate analysis could be reduced. For example, to assess the effective influence of precipitation on surrounding environmental parameters, or the progressive level of deterioration of the landfill’s infrastructure throughout its useful life.

Despite the limitation of the present study, such as not considering the leachate flow in the estimated flow of the DSB, it uses methods applied and recognized as globally efficient. This makes it possible to obtain at least a possible overview of the influence of precipitation on leachate, especially in countries with regions with few hydrological studies. For these reasons, the scarcity of information did not allow the application of new models to estimate the generation of leachate and the comparison with observed data. In addition, there are few similar studies in the Amazon, limiting comparative assessments on what would be the main influences of these parameters in similar environments and what would be the risks and impacts observed on the surrounding populations.

##  Supplemental Information

10.7717/peerj.14686/supp-1 Supplemental Information 1 Variation in potential evapotranspiration (ETo)

10.7717/peerj.14686/supp-2 Supplemental Information 2 Historical Total Monthly Rainfall Hydrograms (mm), A) Fazendinha (Fz), B) Macapá (Mp), C) Sanitary Landfill (LF), D) Porto Grande (PG). Dotted lines (...) = minimum and maximum values. Straight line (––) = mean precipitation historical series. Hydrograms present total monthly rainfall information of Stations Fz (1968–2018), Mp (2014–018), ASMM - LF (2010 –2018) and PG (2008–2015).

10.7717/peerj.14686/supp-3 Supplemental Information 3 Double-mass curve of completed and extended rainfall (mm) It presented the lowest distortion and the best regression parameters. With respect to the other stations, and based on the double mass graphic, one can see the series’ reliability, because the association between mean annual values and annual precipitation accumulation values showed correlation coefficients close to 1.00 in each station. Fz was the most reliable station to feature precipitation in the study site: R2 = 0.999, without significant variation in the historical series (consistence and 50-year continuity). The smallest difference was observed between Fz and LF.

10.7717/peerj.14686/supp-4 Supplemental Information 4 Rainfall period 1958 - 2018 (a) Mean monthly rainfall and (b) Completed and extended rainfall variability (mm) of the considered rainfall gauge stations. The most relevant result was the greatest difference between Fz and Mp variability.

10.7717/peerj.14686/supp-5 Supplemental Information 5 Hydrograms in total monthly rainfall (mm) box-plot per gauges The box-plot diagrams of Pm showed the same. The rainy season starts in December, and it slowly increases Pm until it reaches its maximum values in March and April. After this period Pm starts to decrease until it reaches its minimum values between September and November. Values close to, or within, the very LF station were taken into account to feature Pm.

10.7717/peerj.14686/supp-6 Supplemental Information 6 Climatic variables to estimate potential evapotranspiration Mean monthly temperature (Tx_*m*_) ranges from 26.1° C to 28.9° C and its annual variation records 27.5° C. Ur varies from 70% to 86%, and the highest values are recorded between January and May; its annual mean reaches 79%. W v ranges from 1.4 to 2.7m s ^−1^, and it records annual mean of 1.9 m s^−1^. August and December are the months with the highest Tx _*m*_, they presented high Wv and ETo. Mean ETo was 1,493.76 mm month^−1^. In these cases, ETo represents ≈ 65% of the total precipitation.

10.7717/peerj.14686/supp-7 Supplemental Information 7 Summary of statistical analysis results of historical monthly total rainfall records (mm) in the three stations (data sampling) The time periods were analyzed through statistical methods (Student’s *t* and F-Fisher tests) by comparing the reliable period (scarce series) and by identifying periods extrapolating the confidence interval (95%), or the time when there were potential significant changes in the trends. There were significant changes in periods 1983, 1984-1986 (Fz); 2013, 2015 (Mp); 2010-2011 (LF); and 2014-2018 (PG). In these cases, it would imply data inconsistency that demanded correction.

10.7717/peerj.14686/supp-8 Supplemental Information 8 Summary of statistical analysis results of completed and extended monthly total rainfall records The Fz station recorded the longest temporal series of precipitation data in Amapá State (50 years), and it was used as reference for the other stations –the non-informed sequences in the stations in the same period were fulfilled, as long as they did not present significant variations

10.7717/peerj.14686/supp-9 Supplemental Information 9 Completed and extended (mm) mean multi-year monthly rainfall of the considered rainfall stations (1968–2018 series) Once the regression and correlation methods were applied to precipitation historical data extrapolation, it was possible finding consistency in records of the four stations in the analysis, as well as treating rainfall information

10.7717/peerj.14686/supp-10 Supplemental Information 10 Matapi Basin features and morphometric parameters Values of characterization of DSB.

10.7717/peerj.14686/supp-11 Supplemental Information 11 Maximum rainfall within 24 h recorded for different return times

10.7717/peerj.14686/supp-12 Supplemental Information 12 Comparison between area flow (PMS-01) and leachate flow generation based on the Swiss and rational methods

## Additional Information and Declarations

### Data Availability

The following information was supplied regarding data availability: The raw data is available in the Supplemental Files.
