# Supplementary figures and images for: Hydrometeorological characterization and estimation of landfill leachate generation in the Eastern Amazon/Brazil
Source: PeerJ. 2023 Jan 23;11:e14686. doi: 10.7717/peerj.14686 (PMC9879154; doi:10.7717/peerj.14686)

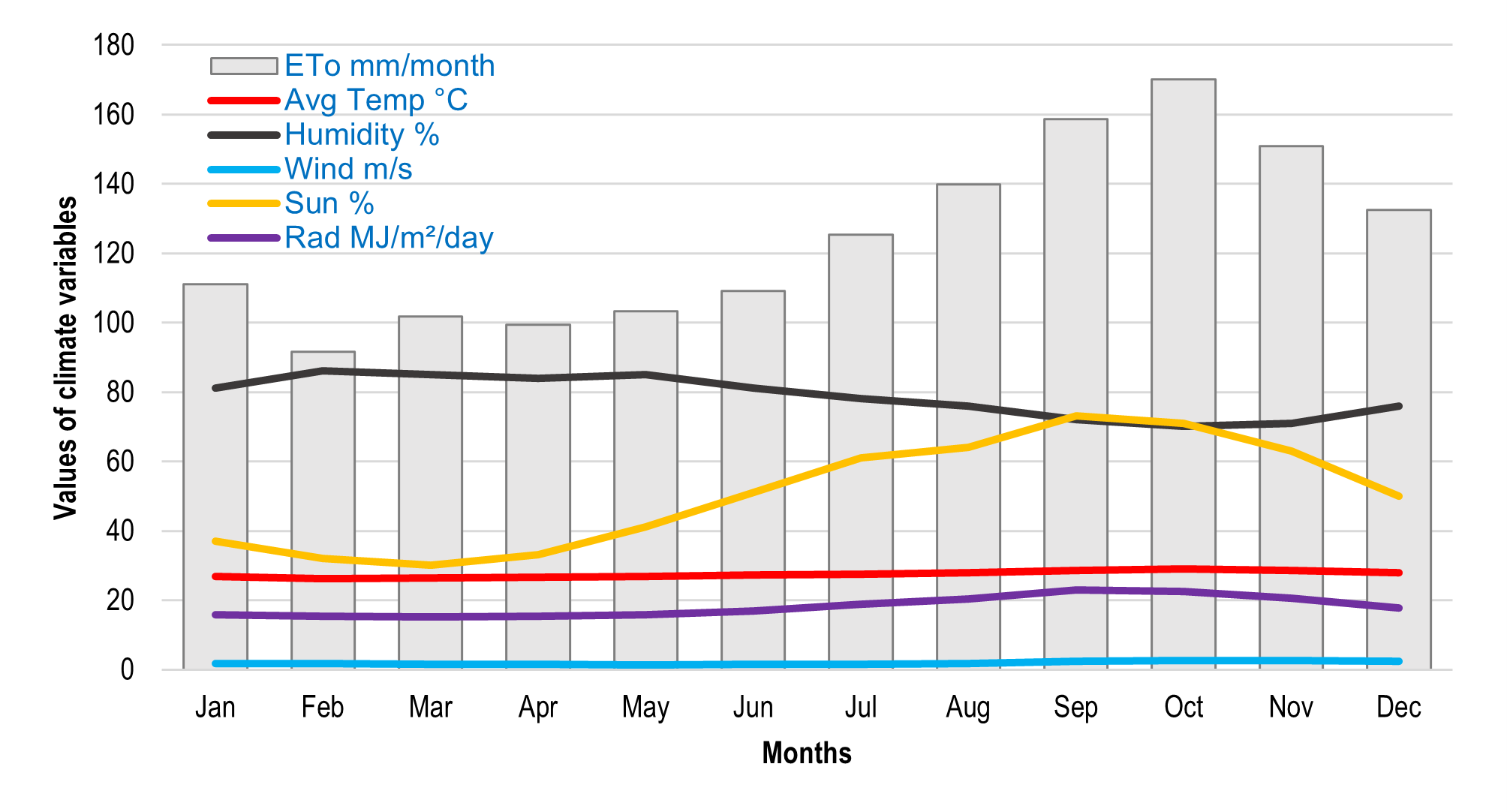

Supplement: Supplemental Information 1 [file peerj-11-14686-s001.png]

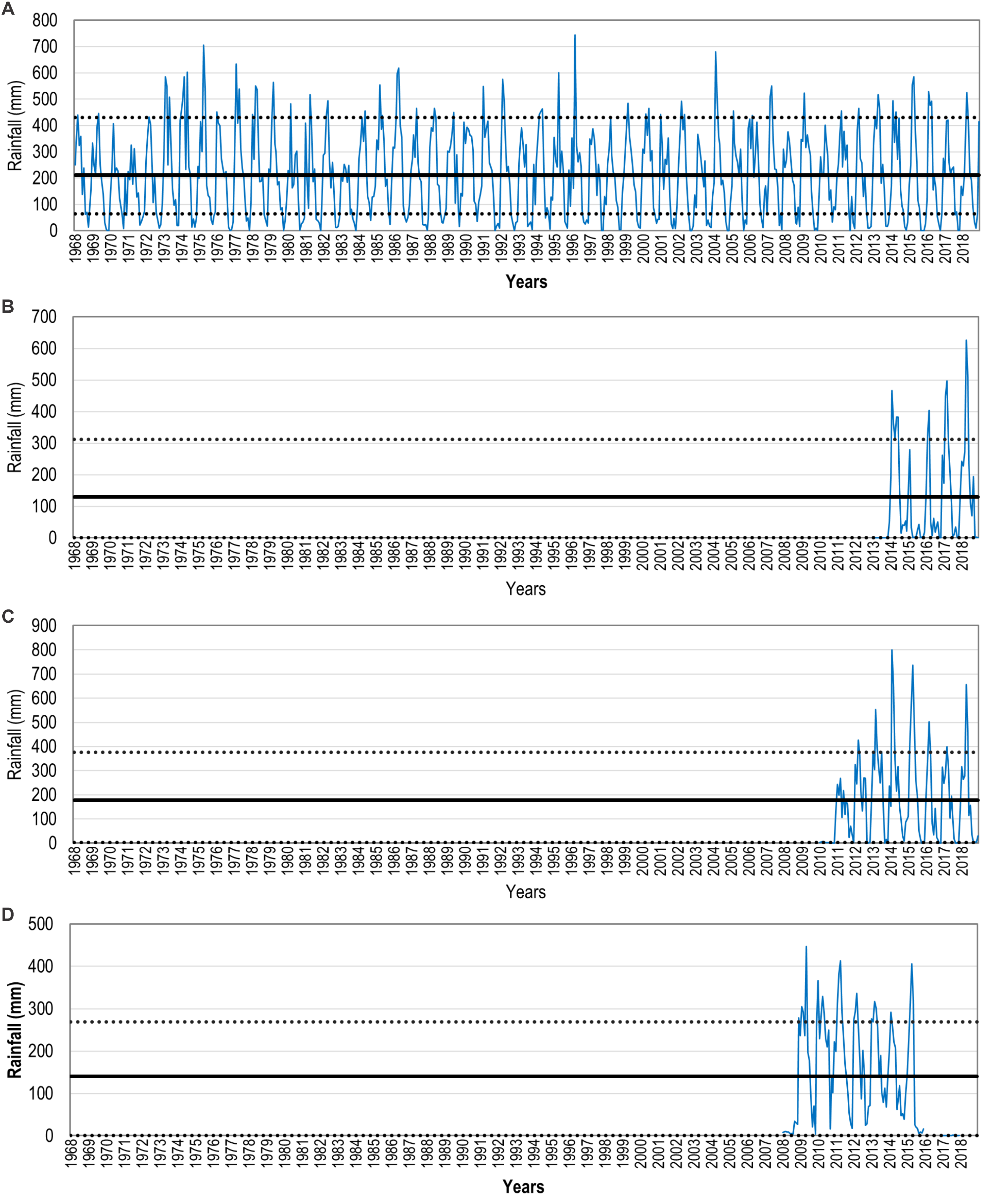

Supplement: Supplemental Information 2 — A) Fazendinha (Fz), B) Macapá (Mp), C) Sanitary Landfill (LF), D) Porto Grande (PG). Dotted lines (...) = minimum and maximum values. Straight line (––) = mean precipitation historical series. Hydrograms present total monthly rainfall information of Stations Fz (1968–2018), Mp (2014–018), ASMM - LF (2010 –2018) and PG (2008–2015). [file peerj-11-14686-s002.png]

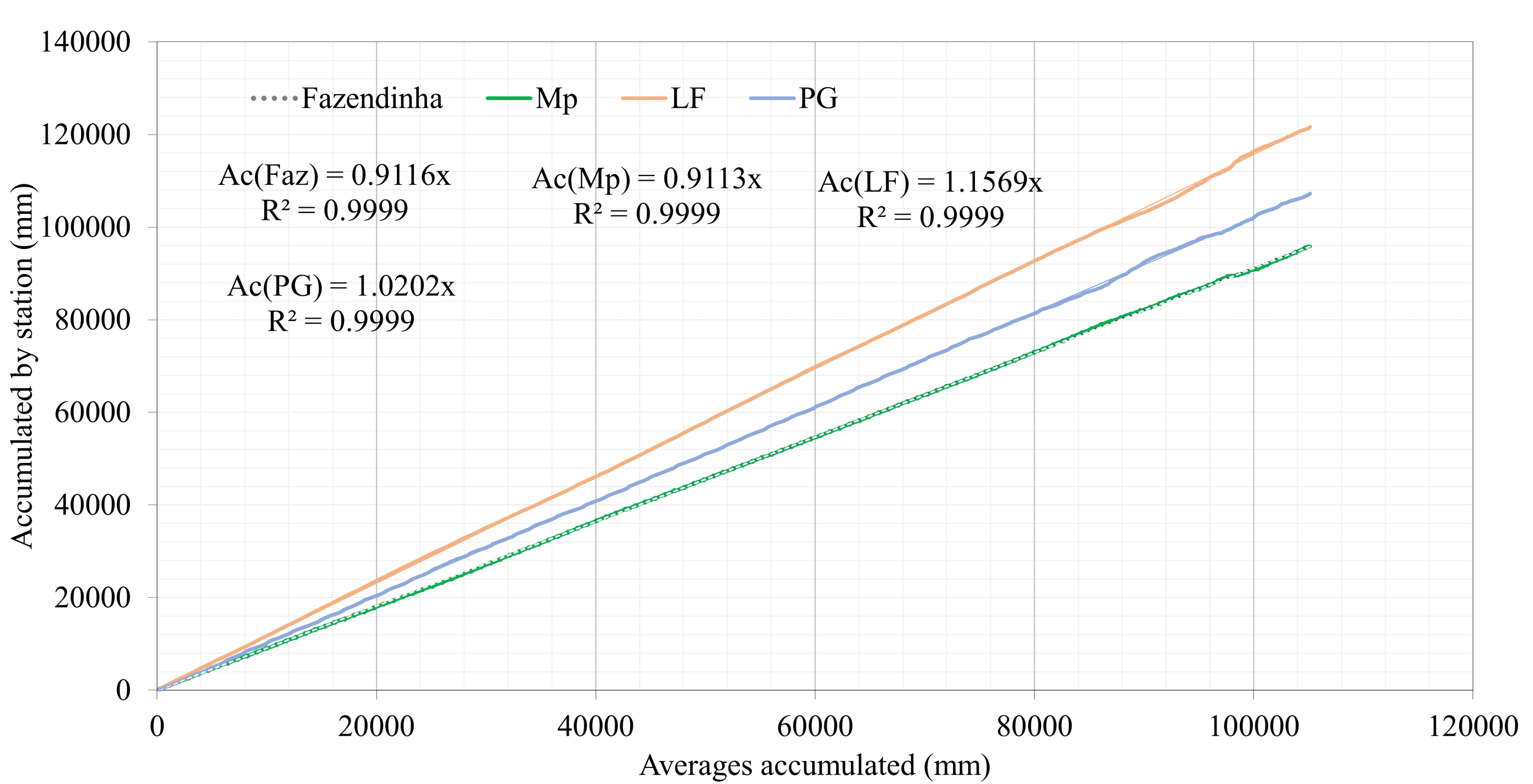

Supplement: Supplemental Information 3 — It presented the lowest distortion and the best regression parameters. With respect to the other stations, and based on the double mass graphic, one can see the series’ reliability, because the association between mean annual values and annual precipitation accumulation values showed correlation coefficients close to 1.00 in each station. Fz was the most reliable station to feature precipitation in the study site: R2 = 0.999, without significant variation in the historical series (consistence and 50-year continuity). The smallest difference was observed between Fz and LF. [file peerj-11-14686-s003.png]

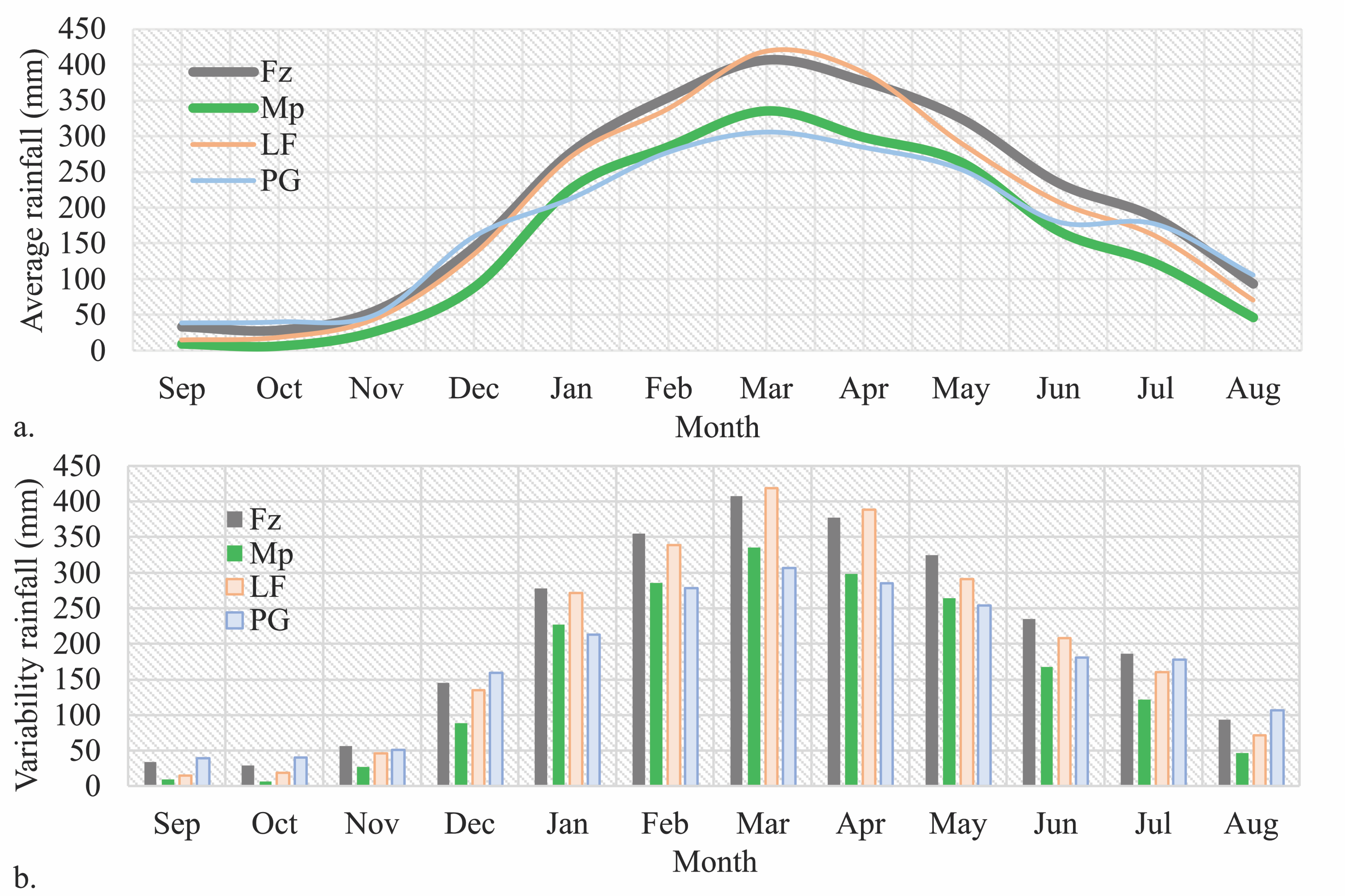

Supplement: Supplemental Information 4 — (a) Mean monthly rainfall and (b) Completed and extended rainfall variability (mm) of the considered rainfall gauge stations. The most relevant result was the greatest difference between Fz and Mp variability. [file peerj-11-14686-s004.png]

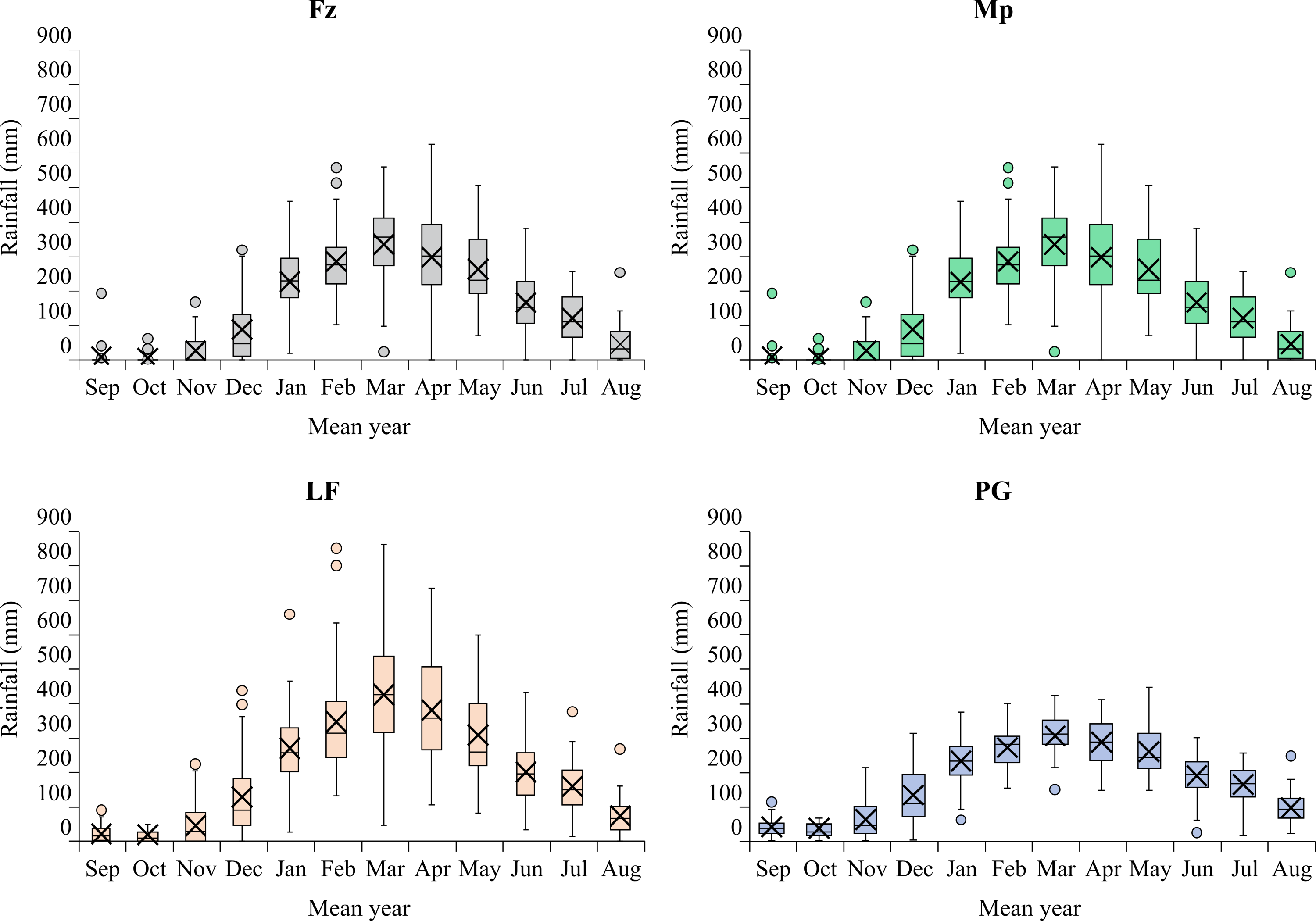

Supplement: Supplemental Information 5 — The box-plot diagrams of Pm showed the same. The rainy season starts in December, and it slowly increases Pm until it reaches its maximum values in March and April. After this period Pm starts to decrease until it reaches its minimum values between September and November. Values close to, or within, the very LF station were taken into account to feature Pm. [file peerj-11-14686-s005.png]
