# Supplementary material for: Hydrometeorological characterization and estimation of landfill leachate generation in the Eastern Amazon/Brazil
Source: PeerJ. 2023 Jan 23;11:e14686. doi: 10.7717/peerj.14686 (PMC9879154; doi:10.7717/peerj.14686)
Supplement: Supplemental Information 6 — Mean monthly temperature (Txm) ranges from 26.1° C to 28.9° C and its annual variation records 27.5° C. Ur varies from 70% to 86%, and the highest values are recorded between January and May; its annual mean reaches 79%. W v ranges from 1.4 to 2.7m s −1, and it records annual mean of 1.9 m s−1. August and December are the months with the highest Tx m, they presented high Wv and ETo. Mean ETo was 1,493.76 mm month−1. In these cases, ETo represents ≈ 65% of the total precipitation. [file peerj-11-14686-s006.docx]

Table S2. Climatic variables to estimate potential evapotranspiration.

| Month | Tx_m_ | Ur | Wv | Sun | Rs | ETo |
| --- | --- | --- | --- | --- | --- | --- |
|  | °C | % | m s^-1^ | % | MJ m^-2^ day^-1^ | mm Month^-1^ |
| Jan | 26.9 | 81 | 1.8 | 37 | 15.7 | 111.04 |
| Feb | 26.1 | 86 | 1.7 | 32 | 15.3 | 91.59 |
| Mar | 26.4 | 85 | 1.6 | 30 | 15.1 | 101.69 |
| Apr | 26.6 | 84 | 1.5 | 33 | 15.3 | 99.42 |
| May | 26.8 | 85 | 1.4 | 41 | 15.8 | 103.23 |
| Jun | 27.3 | 81 | 1.5 | 51 | 16.9 | 109.06 |
| Jul | 27.5 | 78 | 1.5 | 61 | 18.8 | 125.28 |
| Aug | 28.0 | 76 | 1.8 | 64 | 20.4 | 139.72 |
| Sep | 28.5 | 72 | 2.4 | 73 | 22.9 | 158.70 |
| Oct | 28.9 | 70 | 2.7 | 71 | 22.5 | 170.18 |
| Nov | 28.6 | 71 | 2.5 | 63 | 20.6 | 150.95 |
| Dec | 27.9 | 76 | 2.3 | 50 | 17.8 | 132.52 |
| Average | 27.5 | 79 | 1.9 | 51 | 18.1 | 1,493.76 |

Source: Elaborated by the authors. Data gathered from climatic information analyses.
