# Supplementary material for: Hydrometeorological characterization and estimation of landfill leachate generation in the Eastern Amazon/Brazil
Source: PeerJ. 2023 Jan 23;11:e14686. doi: 10.7717/peerj.14686 (PMC9879154; doi:10.7717/peerj.14686)
Supplement: Supplemental Information 7 — The time periods were analyzed through statistical methods (Student’s t and F-Fisher tests) by comparing the reliable period (scarce series) and by identifying periods extrapolating the confidence interval (95%), or the time when there were potential significant changes in the trends. There were significant changes in periods 1983, 1984-1986 (Fz); 2013, 2015 (Mp); 2010-2011 (LF); and 2014-2018 (PG). In these cases, it would imply data inconsistency that demanded correction. [file peerj-11-14686-s007.docx]

Table S3. Summary of statistical analysis results of historical monthly total rainfall records (mm) in the three stations (data sampling)

| Meteorological Station | Analysis Periods | N | $\overline{X}$ | S | Statistical test 95% | | Fc | Ft | Change | |
| --- | --- | --- | --- | --- | --- | --- | --- | --- | --- | --- |
|  |  |  |  |  | Tc | Tt |  |  | $\overline{X}$ | S |
| Fazendinha (Fz) | 1968-1970 | 36.00 | 187.31 | 132.95 | 0.563 | 1.656 | 1.317 | 1.639 | NO | NO |
|  | 1971-1975 | 60.00 | 223.99 | 183.94 | 0.780 | 1.654 | 0.688 | 1.516 | NO | NO |
|  | 1976-1982 | 84.00 | 209.38 | 167.39 | 0.260 | 1.653 | 0.831 | 1.417 | NO | NO |
|  | 1983 | 12.00 | 135.55 | 93.41 | 1.505 | 1.658 | 2.669 | 2.467 | NO | YES |
|  | 1984-1986 | 36.00 | 259.98 | 166.30 | 1.885 | 1.656 | 0.639 | 1.639 | YES | NO |
|  | 1987-1992 | 72.00 | 217.00 | 159.76 | 0.577 | 1.654 | 0.912 | 1.444 | NO | NO |
|  | 1993-1996 | 48.00 | 211.16 | 168.80 | 0.285 | 1.655 | 0.817 | 1.541 | NO | NO |
|  | 1997-2005 | 108.00 | 108.00 | 152.59 | - | - | - | - | - | - |
|  | 2006-2014 | 108.00 | 211.89 | 153.31 | 0.410 | 1.641 | 0.991 | 1.383 | NO | NO |
|  | 2015-2018 | 48.00 | 206.50 | 1976.00 | 0.114 | 1.655 | 0.777 | 1.541 | NO | NO |
| Macapá  (Mp) | 2013 | 12.00 | 4.28 | 14.34 | 4.017 | 1.717 | 140.287 | 2.820 | YES | YES |
|  | 2014 | 12.00 | 201.87 | 169.80 | - | - | - | - | - | - |
|  | 2015 | 12.00 | 44.80 | 83.75 | 2.874 | 1.717 | 4.111 | 2.820 | YES | YES |
|  | 2016-2017 | 24.00 | 136.01 | 156.06 | 1.160 | 1.691 | 1.184 | 2.240 | NO | NO |
|  | 2018 | 12.00 | 207.33 | 197.48 | 0.073 | 1.717 | 0.739 | 2.820 | NO | NO |
| Landfill  (LF) | 2010-2011 | 24.00 | 74.23 | 91.49 | 2.833 | 1.666 | 5.124 | 1.894 | YES | YES |
|  | 2012-2014 | 24.00 | 225.66 | 158.61 | 0.532 | 1.666 | 1.705 | 1.894 | NO | NO |
|  | 2015-2016 | 48.00 | 200.03 | 207.11 | - | - | - | - | - | - |
|  | 2017-2018 | 12.00 | 193.02 | 208.69 | 0.105 | 1.672 | 0.985 | 1.894 | NO | NO |
| Porto Grande  (PG) | 2008-2009 | 36.00 | 150.86 | 130.72 | 1.090 | 1.667 | 0.718 | 1.768 | NO | NO |
|  | 2010-2013 | 36.00 | 182.00 | 110.79 | - | - | - | - | - | - |
|  | 2014-2018 | 36.00 | 89.00 | 117.09 | 3.462 | 1.667 | 0.895 | 1.768 | YES | NO |
