# Supplementary material for: Hydrometeorological characterization and estimation of landfill leachate generation in the Eastern Amazon/Brazil
Source: PeerJ. 2023 Jan 23;11:e14686. doi: 10.7717/peerj.14686 (PMC9879154; doi:10.7717/peerj.14686)
Supplement: Supplemental Information 8 — The Fz station recorded the longest temporal series of precipitation data in Amapá State (50 years), and it was used as reference for the other stations –the non-informed sequences in the stations in the same period were fulfilled, as long as they did not present significant variations [file peerj-11-14686-s008.docx]

Table S4. Summary of statistical analysis results of completed and extended monthly total rainfall records

| Meteorological Station | Analysis Periods | N | $\overline{X}$ | S | Statistical test 95% | | Fc | Ft | Change | |
| --- | --- | --- | --- | --- | --- | --- | --- | --- | --- | --- |
|  |  |  |  |  | Tc | Tt |  |  | $\overline{X}$ | s |
| Fz | 1968-1996 | 348.00 | 211.91 | 160.35 | 0.347 | 1.647 | 0.949 | 1.227 | NO | NO |
|  | 1997-2018 | 264.00 | 207.42 | 156.21 |  |  |  |  |  |  |
| Mp | 1968-1981 | 348.00 | 156.78 | 144.84 | 0.038 | 1.647 | 0.967 | 1.227 | NO | NO |
|  | 2001-2018 | 264.00 | 156.33 | 142.43 |  |  |  |  |  |  |
| LF | 1968-1996 | 348.00 | 200.68 | 175.60 | 0.317 | 1.647 | 0.947 | 1.227 | NO | NO |
|  | 1997-2018 | 264.00 | 196.19 | 170.90 |  |  |  |  |  |  |
| PG | 1965-1989 | 348.00 | 175.83 | 110.64 | 0.169 | 1.647 | 0.974 | 1.227 | NO | NO |
|  | 1990-2017 | 264.00 | 174.31 | 109.18 |  |  |  |  |  |  |
