# Supplementary material for: Hydrometeorological characterization and estimation of landfill leachate generation in the Eastern Amazon/Brazil
Source: PeerJ. 2023 Jan 23;11:e14686. doi: 10.7717/peerj.14686 (PMC9879154; doi:10.7717/peerj.14686)
Supplement: Supplemental Information 9 — Once the regression and correlation methods were applied to precipitation historical data extrapolation, it was possible finding consistency in records of the four stations in the analysis, as well as treating rainfall information [file peerj-11-14686-s009.docx]

Table S5. Completed and extended (mm) mean multi-year monthly rainfall of the considered rainfall stations (1968–2018 series)

| Station | Jan | Feb | Mar | Apr | May | Jun | Jul | Aug | Sep | Oct | Nov | Dec | Total |
| --- | --- | --- | --- | --- | --- | --- | --- | --- | --- | --- | --- | --- | --- |
| Fz | 278.0 | 354.9 | 407.0 | 377.0 | 324.9 | 234.8 | 185.8 | 93.6 | 33.5 | 28.7 | 56.7 | 144.7 | 2,519.7 |
| Mp | 227.1 | 285.7 | 335.5 | 298.6 | 263.7 | 167.5 | 122.0 | 46.5 | 9.5 | 6.6 | 27.6 | 88.6 | 1,879.0 |
| LF | 271.4 | 338.5 | 418.5 | 388.9 | 290.5 | 208.3 | 160.4 | 71.1 | 15.4 | 19.1 | 45.8 | 134.8 | 2,362.7 |
| PG | 213.2 | 278.1 | 306.1 | 284.9 | 254.0 | 180.6 | 177.5 | 106.4 | 39.2 | 40.7 | 51.3 | 159.7 | 2,091.7 |
