# Supplementary material for: Hydrometeorological characterization and estimation of landfill leachate generation in the Eastern Amazon/Brazil
Source: PeerJ. 2023 Jan 23;11:e14686. doi: 10.7717/peerj.14686 (PMC9879154; doi:10.7717/peerj.14686)
Supplement: Supplemental Information 10 — Values of characterization of DSB. [file peerj-11-14686-s010.docx]

Table S1. Matapi Basin features and morphometric parameters.

| Features and morphometric parameters | Unity | Value | Observation |
| --- | --- | --- | --- |
| Area | km^2^ | 11.09 | Small: 25 – 250 |
| Basin perimeter | Km | 18.85 | - |
| Stream length | Km | 5.92 | - |
| Maximum height | m.a.s.l.* | 29.82 | - |
| Minimum height | m.a.s.l.* | 0.97 | - |
| Maximum gradient | m | 28.85 | - |
| Basin longest width | km | 3.50 | - |
| Basin longest length | km | 4.54 | - |
| Compactness coefficient | Dimensionless | 1.60 | Type III: 1.51 - >2 |
| Form factor | Dimensionless | 0.32 | Slightly elongated: 0.30-0.37 |
| Elongation ratio | Dimensionless | 0.83 | Low relief: >8 |
| Mean basin elevation | m | 11.63 | - |
| Mean basin slope | m/m | 0.06 | - |
| Drainage density | km/km^2^ | 0.53 | Low: <1; High: >2 |

*m.a.s.l. is meters above sea level.
