# Supplementary material for: Hydrometeorological characterization and estimation of landfill leachate generation in the Eastern Amazon/Brazil
Source: PeerJ. 2023 Jan 23;11:e14686. doi: 10.7717/peerj.14686 (PMC9879154; doi:10.7717/peerj.14686)
Supplement: Supplemental Information 11 [file peerj-11-14686-s011.docx]

Table S7. Maximum rainfall within 24 hours recorded for different return times

| Station | Distribution model | D_max_ | Return period max (year) (RT) | | | | | |
| --- | --- | --- | --- | --- | --- | --- | --- | --- |
|  |  |  | 2 | 5 | 10 | 25 | 50 | 100 |
| Fz | EV2-Max | 0.0706 | 114.8 | 140.7 | 161.0 | 190.9 | 216.6 | 245.6 |
| Mp | Pearson III | 0.0791 | 94.1 | 142.7 | 169.9 | 200.5 | 221.0 | 240.0 |
| PG | Normal * | 0.0936 | 76.0 | 91.7 | 99.9 | 108.6 | 114.2 | 119.3 |
| Br | Pareto * | 0.0679 | 82.6 | 106.9 | 118.5 | 128.3 | 133.1 | 136.3 |
| Ca | EV2-Max | 0.0414 | 120.6 | 149.9 | 173.2 | 207.8 | 237.9 | 272.1 |
| Be | EV1-Max ** | 0.0721 | 106.5 | 133.3 | 151.1 | 173.5 | 190.1 | 206.6 |
| So | GEV-Min | 0.0354 | 157.5 | 196.8 | 219.5 | 245.0 | 262.0 | 277.7 |
| Tr | GEV-Min * | 0.0819 | 114.4 | 151.1 | 174.1 | 174.1 | 201.2 | 220.0 |
| Al | GEV-Max * | 0.0654 | 112.9 | 148.9 | 175.0 | 210.9 | 239.6 | 270.2 |
| Bl | Pareto * | 0.0810 | 113.9 | 148.6 | 161.7 | 170.6 | 173.9 | 175.8 |
| Ob | EV1-Max ** | 0.0696 | 102.0 | 127.8 | 145.0 | 166.6 | 182.7 | 198.6 |
| MA | Pearson III | 0.0393 | 103.9 | 155.4 | 142.7 | 159.2 | 170.6 | 181.3 |

* L-Moments, ** Gumbel
