# Supplementary material for: Hydrometeorological characterization and estimation of landfill leachate generation in the Eastern Amazon/Brazil
Source: PeerJ. 2023 Jan 23;11:e14686. doi: 10.7717/peerj.14686 (PMC9879154; doi:10.7717/peerj.14686)
Supplement: Supplemental Information 12 [file peerj-11-14686-s012.docx]

Table S9. Comparison between area flow (PMS-01) and leachate flow generation based on the Swiss and rational methods

| Method | Jan | Feb | Mar | Apr | May | Jun | Jul | Aug | Sep | Oct | Nov | Dec | Year |
| --- | --- | --- | --- | --- | --- | --- | --- | --- | --- | --- | --- | --- | --- |
| PMS-01 (m^3^.s^-1^) | 0.041 | 0.047 | 0.046 | 0.045 | 0.038 | 0.033 | 0.028 | 0.014 | 0.006 | 0.004 | 0.004 | 0.018 | 0.027 |
| Swiss  (m^3^.s^-1^) | 0.003 | 0.004 | 0.004 | 0.004 | 0.003 | 0.002 | 0.002 | 0.001 | 0.000 | 0.000 | 0.001 | 0.001 | 0.002 |
| Rational (m^3^.s^-1^) | 0.004 | 0.007 | 0.007 | 0.007 | 0.005 | 0.002 | 0.000 | -0.003 | -0.005 | -0.005 | -0.004 | -0.001 | 0.013 |
